# Zinc dampens antitumor immunity by promoting Foxp3^+^ regulatory T cells

**DOI:** 10.3389/fimmu.2024.1389387

**Published:** 2024-08-23

**Authors:** Sugandha Narayan, Rajdeep Dalal, Zaigham Abbas Rizvi, Amit Awasthi

**Affiliations:** ^1^ Centre for Immunobiology and Immunotherapy, Translational Health Science and Technology Institute, National Capital Region (NCR)-Biotech Science Cluster, Faridabad, Haryana, India; ^2^ Immunology Core Lab, Translational Health Science and Technology Institute, National Capital Region (NCR)-Biotech Science Cluster, Faridabad, Haryana, India

**Keywords:** checkpoint inhibition therapy, zinc, regulatory T cell, cancer, FOXO1, antitumor immunity, PD-1

## Abstract

**Introduction:**

The role of zinc (Zn) in tumor development and immune modulation has always been paradoxical. This study redefines our understanding of the impact of Zn on cancer progression and therapeutic strategies.

**Methods:**

We investigated the effects of dietary Zn levels on tumor progression and immune responses. This included examining the impact of both high and deficient dietary Zn, as well as Zn chelation, on tumor growth and immune cell populations. Specifically, we analyzed the frequency of Foxp3^+^ regulatory T-cells (Tregs) and identified the role of FOXO1 in Zn-mediated effects on Tregs. Additionally, we explored the therapeutic potential of clioquinol (CQ) in enhancing ^α^-PD-1 immunotherapy responses, particularly in melanoma.

**Results:**

Our findings show that high dietary Zn promotes tumor progression by fostering a protumorigenic environment mediated by T cells. Increased Zn intake was found to facilitate tumor progression by increasing Foxp3^+^ Treg frequency. In contrast, deficiency in dietary Zn and chelation of tissue Zn emerged as potent drivers of antitumor immunity. We pinpointed FOXO1 as the master regulator governing the influence of Zn on Tregs.

**Discussion:**

These results reveal a novel mechanistic insight into how Zn influences tumor progression and immune regulation. The identification of FOXO1 as a key regulator opens new avenues for understanding the role of Zn in cancer biology. Furthermore, we introduce a promising therapeutic approach by showing that administering clioquinol (CQ) significantly enhances ^α^-PD-1 immunotherapy response, particularly in melanoma. These revelations transform our comprehension of the multifaceted role of Zn in tumorigenesis and immune regulation, highlighting innovative possibilities for cancer therapy.

## Introduction

Cancer remains a formidable global health challenge, affecting millions of individuals worldwide. As per current WHO estimates, cancer is going to be a major burden on the world economy, with the global economic cost of cancers estimated to be $25.2 trillion from 2020 to 2050 (1). Despite significant advancements in cancer research and treatment modalities, the complexities of tumor biology and the dynamic interplay between the immune system and cancer cells continue to pose major obstacles in achieving long-term remission and improved patient outcomes (2). Cancer treatment employs diverse therapeutic modalities, including surgery, chemotherapy, radiation therapy, targeted therapy, and immunotherapy (3). Immunotherapy, however, is gaining prominence due to its innovative approach. This therapy harnesses the body’s immune system to recognize and attack cancer cells (4). Approaches like immune checkpoint inhibitors, CAR-T cell therapy, and cancer vaccines have demonstrated remarkable success by bolstering immune responses against tumors (5–7). Immunotherapy offers the potential for durable and targeted treatments, marking a transformative advancement in the fight against cancer.

In recent years, the role of trace elements in cancer development and immune regulation has garnered increasing attention (8–10). Micronutrients, such as zinc (Zn) and magnesium (Mg), play a pivotal role in immune regulation, particularly concerning T cells and other immune cells. Zinc is essential for T-cell development, differentiation, and function, as it influences signal transduction pathways and gene expression critical for immune responses (11). Magnesium is crucial for maintaining T-cell proliferation, cytokine production, and activation (12). Additionally, other micronutrients like selenium and vitamin D also contribute to immune cell activities, influencing their maturation, movement, and response to infections (13). Overall, an adequate supply of these micronutrients is vital for a well-balanced immune system, supporting effective immune responses and optimal defense against pathogens (14).

Micronutrients play a crucial role in cancer prevention and treatment due to their impact on cellular function and immune response (15). Vitamins (e.g., A, C, D, E), minerals (e.g., zinc, selenium), and antioxidants are known for maintaining a healthy immune system and reducing oxidative stress, which can contribute to cancer development (14). However, a detailed study is needed to determine their precise effects on various types of cancer. Aligned with this, a preceding study conducted in our laboratory has substantiated that elevated salt intake effectively inhibits tumor growth, primarily through the augmentation of natural killer (NK) cell activity and enhancing the responsiveness to PD-1 therapy (16). Among trace elements, Zn stands out as one of the critical micronutrients, playing essential roles in various physiological processes, including cellular growth, differentiation, and DNA repair (17). However, emerging evidence suggests that Zn’s intricate involvement in tumor biology extends beyond its fundamental cellular functions (18).

Studies exploring the relationship between Zn intake and cancer risk have yielded intriguing and, at times, contradictory findings. Some reports indicated a positive association between high Zn intake and an increased risk of cancer development (19–22), while others have pointed toward the potential protective effects of Zn against certain cancer types (23, 24). The exact mechanisms underlying Zn and its association with cancer progression or regression remain elusive, warranting further investigation into the impact of Zn on tumor growth and immune response. Our particular interest is in the influence of Zn on immune cells within the tumor microenvironment. T cells, in particular, play a pivotal role in mounting an immune response against cancer cells. Regulatory T cells (Treg cells) and PD-1^+^ T cells have been implicated in shaping the immunosuppressive environment of tumors, allowing cancer cells to evade immune detection and destruction (25, 26). Understanding how Zn affects the balance of these immune cell populations within the tumor microenvironment could provide valuable insights into the potential use of Zn modulation in cancer immunotherapy. Moreover, the relationship between Zn intake and response to immunotherapeutic strategies, such as checkpoint blockade therapies, remains poorly explored. Given the remarkable success of checkpoint inhibitors in specific cancers, it becomes crucial to investigate whether Zn levels influence the efficacy of these treatments and, thus, to explore possible strategies to enhance their effectiveness in Zn-rich tumor microenvironments.

In this study, we sought to elucidate the impact of Zn intake on tumor burden and the immune response using syngeneic mouse tumor models. Here, we show that high levels of Zn promote melanoma progression while a zinc-deficient diet and tissue Zn chelation remarkably bolstered antitumor immunity, resulting in significantly reduced tumor volumes. Our study also unveils that elevated zinc intake heightened the presence of Foxp3^+^ Treg cells and upregulated PD-1^+^ T cells within the tumor microenvironment, with T-cell-deficient (RAG1^−/−^) and Foxp3-depleted mice mitigating the tumor-promoting effects of zinc. We also elucidated a novel mechanistic insight by showing the role of FOXO1 in Zn-mediated regulation of Foxp3^+^ Treg cells. Finally, we showed that clioquinol (CQ) administration synergistically enhances α-PD-1 immunotherapy in B16 melanoma. Thus, by investigating the mechanisms through which Zn exerts its protumor effects and how it modulates immune cell function, we aimed to shed light on potential targets for therapeutic intervention and improve our understanding of the intricate relationship between Zn and cancer immunobiology. The findings from this research could contribute significantly to the development of novel approaches for cancer treatment, ultimately leading to better outcomes and improved quality of life for cancer patients.

## Results

### High Zn intake increases tumor burden in syngeneic mouse tumor models

**Figure 1:**
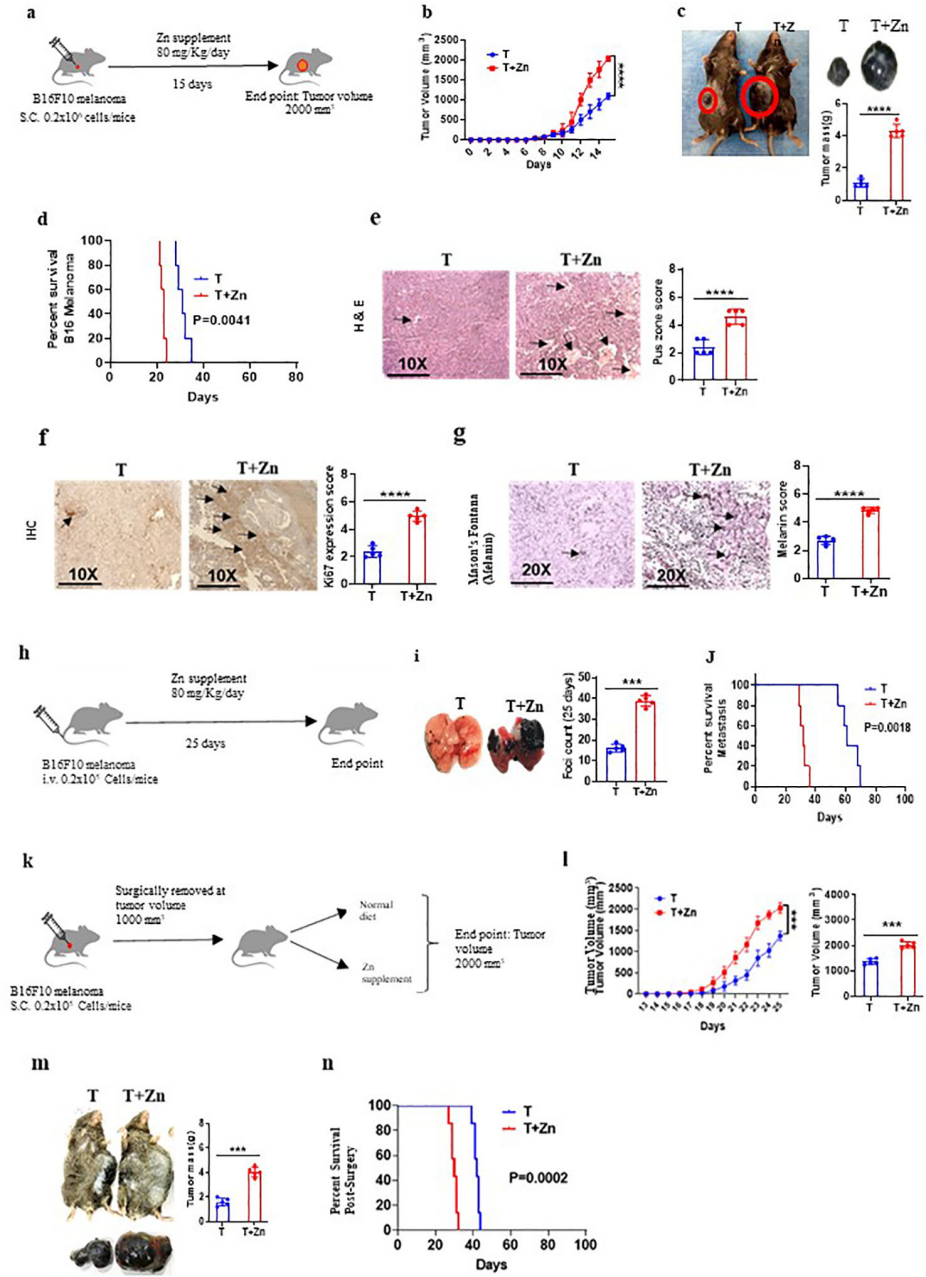
High zinc (Zn) intake increases tumor burden in syngeneic mouse tumor models. **(A)** Schematic representation of the experiment in which B16F10 melanoma was implanted in C57BL/6 mice. While one of the groups was given a Zn supplement of 80 mg/kg/day, the other was kept on a normal diet as the control. **(B)** The rate of growth of tumor volume. **(C)** The tumor mass in the groups of mice consuming a normal diet (T) and high Zn intake (T+Zn). **(D)** The percent survival of mice bearing B16F10 melanoma consuming a normal diet (T) vs. high Zn intake (T+Zn). **(E–G)** Histology of the tumor tissue samples: hematoxylin and eosin (H&E) stain (×10) to identify pus zones **(E)**, immunohistochemistry (IHC) (×10) to identify Ki67 expression **(F)**, and Masson’s Fontana stain (×20) to identify the level of melanin production **(G)**. **(H)** Schematic representation of the metastasis model in which B16F10 cells were injected intravenously into the tail vein and given Zn supplement for one group of mice (T+Zn) while the other group was kept on a normal diet as the control (T). **(I)** The foci count on the lungs of T vs. T+Zn. **(J)** The percent survival of metastasis in group T vs. group T+Zn. **(K)** Schematic representation of the surgical model experiment in which tumor from mice was surgically removed at 1,000 mm^3^ and then the animals were put either on a normal diet (T) or a high Zn diet (T+Zn). **(L)** Rate of growth of tumor volume post-surgery. **(M)** Post-surgery tumor recurrence in group T vs. group T+Zn. **(N)** The post-surgery percent survival rate in the Zn-supplemented group compared with the control group consuming a normal diet. Data are representative of the mean ± SEM from three independent experiments. *P < 0.05, **P < 0.01, ***P < 0.001, and ****P < 0.0001 (Student’s *t*-test or one-way ANOVA); percent survival by the Mantel–Cox test; number of animals (*n* = 7) in all experiments.

To understand the effect of high Zn intake on tumor growth, we implanted B16F10 melanoma in C57BL/6 mice. While one of the groups was given a Zn supplement of 80 mg/kg/day, the other group was kept on a normal diet (AIN76A) as the control (**Figure 1A**). We used zinc sulfate heptahydrate (ZnSO_4_ 7H_2_O) as an oral Zn supplement with an LD50 value of 926 mg/kg in mice (27). According to AIN76A, the normal chow diet of mice contains approximately 30 ppm of Zn (28). We did not observe any toxicity by using a Zn supplement of 80 mg/kg/day in mice. The parameters that were evaluated in order to thoroughly evaluate the potential toxicity of the Zn treatment are described in the *Materials and methods* section (**Supplementary Figures 1**, **2**). All the experiments were reproduced independently at least three times, ensuring the reliability and robustness of our findings with the number of animals (*n* = 7) in each experimental setup. The tumor volume was measured every alternative day until it reached 2,000 mm^3^. The rate of growth of tumor volume was high in the group of mice consuming high Zn (T+Zn), compared to the control group on normal diet (T) (**Figure 1B**). The tumor mass was also found to be higher in the T+Zn group than in the T group (**Figure 1C**). We further tested the effect of high Zn intake on the survival of B16F10 melanoma-bearing C57BL/6 mice and found that the Zn-supplemented group had a lesser survival rate with high mortality as compared to the control group (**Figure 1D**). In line with this, we also used high dietary Zn as a presupplement in which mice were kept on high dietary Zn before tumor implantation and switched to a normal laboratory diet after tumor implantation (**Supplementary Figure 3A**). We found that prior treatment with high dietary Zn increased tumor growth. The pre-Zn-supplemented groups showed a high rate of tumor growth and high tumor mass compared to the group consuming a normal diet (**Supplementary Figure 3B**). The survival study showed that the pre-Zn-supplemented group had a lesser survival rate compared to the control group consuming a normal diet (**Supplementary Figure 3C**). The histology of the tumor tissue samples revealed that the group of mice consuming high Zn (T+Zn) showed elevated levels of pus zones (H&E staining) (**Figure 1E**), a higher level of Ki67 expression (immunohistochemistry, IHC) (**Figure 1F**), and a higher level of melanin production (Masson’s Fontana staining) (**Figure 1G**) compared to the control group consuming a normal diet (T). The presence of pus zones, also known as necrosis, can indicate tumor burden and aggressiveness. The presence of large necrotic (pus zones) within a tumor can indicate a larger tumor size and an aggressive phenotype (29). Ki67 is a protein found in the nucleus of cells and is associated with cell division and proliferation. A higher level of Ki67 expression indicates that a larger proportion of cells are actively dividing, suggesting a more aggressive tumor with potentially faster growth (30). Melanin, the pigment in the skin, can indicate aggressive cancer, especially in melanoma. Higher melanin production in these tumors may suggest increased cellular activity and proliferation, traits of a more aggressive cancer (31).

Since a high Zn diet has been shown to be associated with tumor progression, we tested the effect of high dietary Zn on tumor metastasis. In this study, we chose the B16F10 intravenous cell injection model for its well-established and widely accepted use as a model for studying tumor metastasis (16, 32, 33). To do this, B16F10 cells were injected intravenously into the tail vein, and a Zn supplement was given to one group of mice, while another group was kept on a normal diet as the control (**Figure 1H**). Our data indicated that the group of mice which was on high dietary Zn showed an excess number of tumor foci on the lungs as compared to the control group (**Figure 1I**). The survival study showed that metastasis-induced mice consuming high levels of Zn had a lesser survival rate as compared to the mice consuming a normal diet (**Figure 1J**). We also tested the role of high Zn intake in a surgical model of a tumor to test post-surgery tumor recurrence. We implanted B16F10 melanoma in C57BL/6 mice, and once the tumor volume reached 1,000 mm^3^, it was surgically removed and the mice were kept on either a normal diet or Zn supplement post-surgery and monitored for tumor recurrence (**Figure 1K**). The post-surgery tumor recurrence was measured every alternative day until the volume reached 2,000 mm^3^. The rate of tumor recurrence was high in the Zn-supplemented group (T+Zn) as compared to the control group (T) (**Figure 1L**). The post-surgical recurrence of tumor mass was high in the T+Zn group as compared to the T group (**Figure 1M**). In line with this, the post-surgery survival rate was lower in the Zn-supplemented group as compared with the control group consuming a normal diet (**Figure 1N**). Taken together, our data demonstrated the role of Zn in tumor progression in different experimental models.

### Zn mediates a pro-tumor effect via T cells

**Figure 2:**
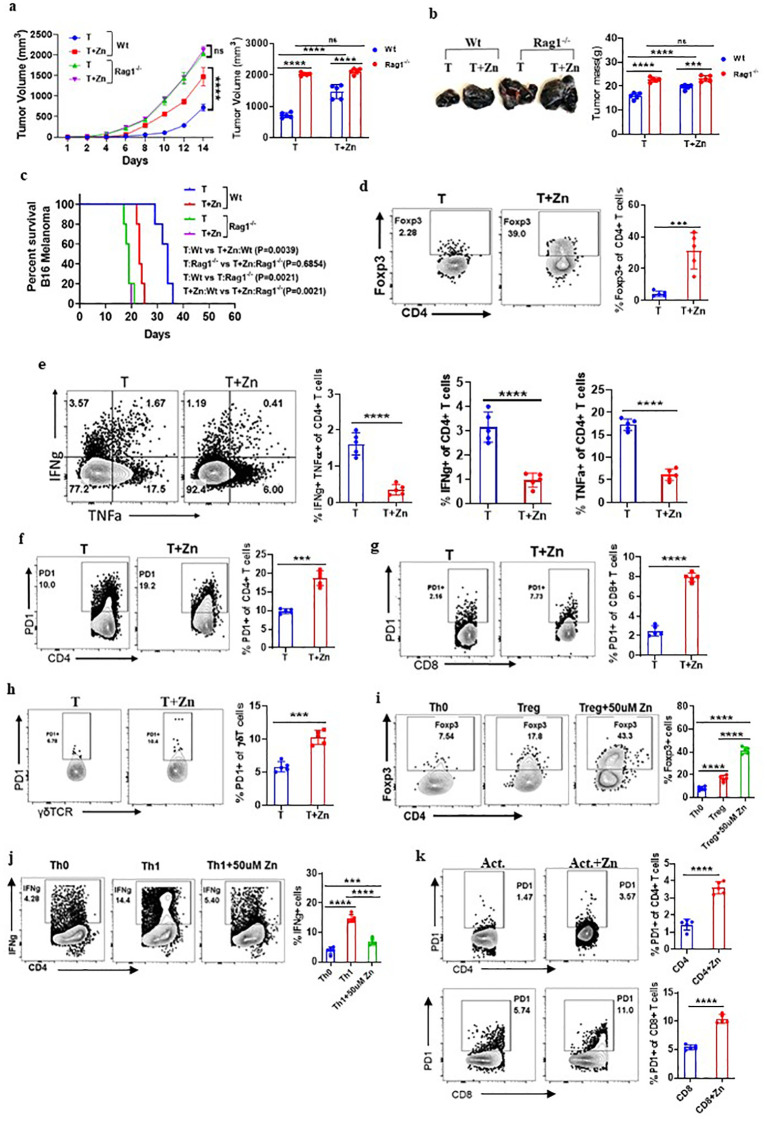
Zn mediates the protumor effect via T cells. **(A)** B16F10 melanoma was implanted in C57BL/6 mice (Wt) and RAG1^−/−^ mice consuming a normal diet (T) and a high Zn diet (T+Zn). The rate of tumor progression is measured every alternative day until the volume reached 2,000 mm^3^. **(B)** The endpoint tumor mass. **(C)** Percent survival curve of B16F10 melanoma-bearing C57BL/6 mice (Wt) vs. RAG1^−/−^ mice consuming a normal diet (T) and a high Zn diet (T+Zn). **(D)** Immunophenotyping of Foxp3^+^ Treg cells (regulatory T cells) from the tumor-infiltrated lymphocytes (TILs) of B16F10 melanoma-bearing C57BL/6 mice consuming a normal diet (T) and high Zn intake (T+Zn). **(E)** Immunophenotyping of IFN-γ^+^ TNF-α^+^ CD4^+^ T (Th1) cells from the TILs of T and T+Zn. **(F–H)** Immunophenotyping of PD-1^+^ CD4^+^ T cells, PD-1^+^ CD8^+^ T cells, and PD-1^+^ γδTCR^+^ T cells from the TILs of T and T+Zn. **(I, J)** Naive CD4^+^ T cells and CD8^+^ T cells are sorted from the spleen of healthy C57BL/6 mice and activated/differentiated *in vitro* in the presence or absence of Zn supplement (50 μM) into Treg cells and Th1 cells. **(K)** Naive CD4^+^ T cells and CD8^+^ T cells are sorted from the spleen of healthy C57BL/6 mice and activated *in vitro* with α-CD3 and α-CD28 antibodies for 48–72 h with or without Zn (50 μM) supplement and measured surface expression of PD-1. Data are representative of the mean ± SEM from three independent experiments. *P < 0.05, **P < 0.01, ***P < 0.001, and ****P < 0.0001 (Student’s *t*-test or one-way ANOVA); percent survival by the Mantel–Cox test; number of animals (*n* = 7) in all experiments.

Our data suggested the role of Zn in promoting tumor growth; however, it is not clear whether adaptive or innate immune cells mediate the protumor effects of Zn. The role of adaptive and innate immune cells was implicated in contributing to tumor growth. It was earlier suggested that Foxp3^+^ Tregs and tumor-associated macrophages (TAMs) were found to be present in the tumor microenvironment (TME) and suggested to support tumor growth by suppressing antitumor immune response (34, 35). To determine the role of the innate and adaptive component of the immune system in Zn-mediated tumor progression, we used RAG1^−/−^ and C57BL/6 WT mice, in which B16F10 melanoma cells were implanted. These mice were kept on normal or high Zn diet, and progression was followed by measuring tumor volume every alternate day until the tumor volume reached 2,000 mm^3^. As shown previously, the tumor growth was found to be increased in RAG1^−/−^ as compared to WT mice, which could be due to the absence of adaptive immune response, especially the antitumor T-cell response (16). We show that while Zn supplementation increases tumor growth, as indicated by tumor mass, in WT mice, it failed to do so in RAG1^−/−^ mice (**Figures 2A, B**). These observations indicated the involvement of adaptive immune cells in Zn-mediated tumor progression. In line with this, our data showed that the survival rate of RAG1^−/−^ mice was not altered with or without the Zn supplement, as opposed to the Zn supplement in C57BL/6 WT mice (**Figure 2C**).

The role of B cells has a limited direct impact on solid tumors (36); therefore, we focused our interest on identifying the subtypes of T lymphocytes that are involved in Zn-mediated tumor progression. To do this, we isolated lymphocytes from the spleen, lymph nodes, and tumor microenvironment from Zn-supplemented and control mice and performed immunophenotyping. We looked into all the types of major immune cells in tumor-infiltrated lymphocytes. We have included a comprehensive gating strategy outlining the approach to analyze the data (**Supplementary Figures 4A, B**). The total number of tumor leukocyte infiltrations remained consistent between the treated and untreated groups (**Supplementary Figure 4C**). However, we found that two major subtypes of CD4 T-cell populations were being altered. The frequency of Foxp3^+^ Treg cells was increased (**Figure 2D**), while the frequency of CD4^+^ IFN-γ^+^ (T helper 1) cells is decreased (**Figure 2E**). We further tested how Zn suppresses the antitumor immune response and performed immunophenotyping of major immune checkpoint molecules such as PD-1, CTLA-4, Tim3, TIGIT, and other immunomodulatory molecules like CD96 and CD107a/LAMP1. We found that Zn supplementation increases the expression of PD-1 on CD4 T cells, CD8 T cells, and γδT cells (**Figures 2F–H**), without affecting the PD-1 expression on NK cells and macrophages (**Supplementary Figure 5**). Interestingly, Zn supplementation decreases the expression of CD107a/LAMP1 on NK cells and CD8 T cells (**Supplementary Figure 6A**). CD107a/LAMP1, known as a degranulation marker, plays an important role in the cytotoxic functions of mature NK and CD8 T cells (37). In line with this, the relative mRNA expression of CD107a was also found to be decreased upon zinc supplementation, as compared to a normal diet, in mice (**Supplementary Figure 6B**). We did not see any difference in the expression levels of CTLA-4, Tim-3, TIGIT, and CD96 on immune cells in the Zn-supplemented or non-Zn-supplemented group of mice (**Supplementary Figure 7**).

The tumor microenvironment involves multiple factors that might be involved directly or indirectly in Zn-mediated modulation of their phenotypes and functions. To test whether Zn directly influences the functions of T cells, we performed an *in-vitro* experiment in which naive CD4^+^ T cells and CD8^+^ T cells were sorted from the spleen of healthy C57BL/6 mice as shown in **Supplementary Figures 8A, B** and activated/differentiated into Treg and Th1 cells with or without Zn (50 μM) supplement. Naive T cells cultured with TGF-β1 in the presence of Zn showed a higher level of *in-vitro* Treg differentiation (**Figure 2I**). Moreover, Zn suppressed *in-vitro* Th1 differentiation (**Figure 2J**), supporting our *in-vivo* finding in tumor settings in which Zn enhanced and suppressed Treg and Th1 cells, respectively. To further test whether Zn supplementation directly impacts the expression of PD-1, as it does so *in vivo* in tumor-bearing mice, the sorted CD4^+^ T cells and CD8^+^ T cells from the spleen of healthy C57BL/6 mice were activated with α-CD3 and α-CD28 antibodies for 48–72 h with or without Zn (50 μM) supplementation. Interestingly, the Zn supplement increased the surface expression of PD-1 on both CD4^+^ and CD8^+^ T cells as compared to the control group (**Figure 2K**). Similarly, we isolated splenocytes from healthy C57BL/6 mice, stimulated with α-CD3 alone with two different concentrations of Zn (15 μM and 50 μM). Our data showed that Zn supplementation increased the surface expression of PD-1 in a dose-dependent manner (**Supplementary Figure 9**). Since PD-1 expression is linked to TcR-dependent activation of T cells, it may be possible that Zn may somehow modulate TcR signaling and enhance PD-1 expression. To rule out this possibility, we gave the first round of activation of splenocytes with α-CD3 antibody for 48–72 h and rested them; subsequently, cells were washed with PBS and cultured with or without Zn supplementation for 48–72 h without the second round of activation. Our data showed that the higher the level of Zn in the media, the higher the PD-1 expression (**Supplementary Figure 10**), suggesting that Zn may not directly play a role in the TCR activation-mediated PD-1 overexpression. Since our *in-vivo* data indicated that Zn supplementation in tumor settings downregulates CD107a, we therefore tested the effect of Zn supplementation on CD107a of stimulated NK cells and CD8 T cells. We showed that both NK and CD8 T cells downregulated the expression of CD107 by Zn supplementation as compared to the control (**Supplementary Figure 11**). Altogether, these data indicated the immunomodulatory role of Zn in the tumor microenvironment by altering the T-cell phenotypes and functions.

### Dietary Zn deficiency and tissue Zn chelation promote antitumor immunity

**Figure 3:**
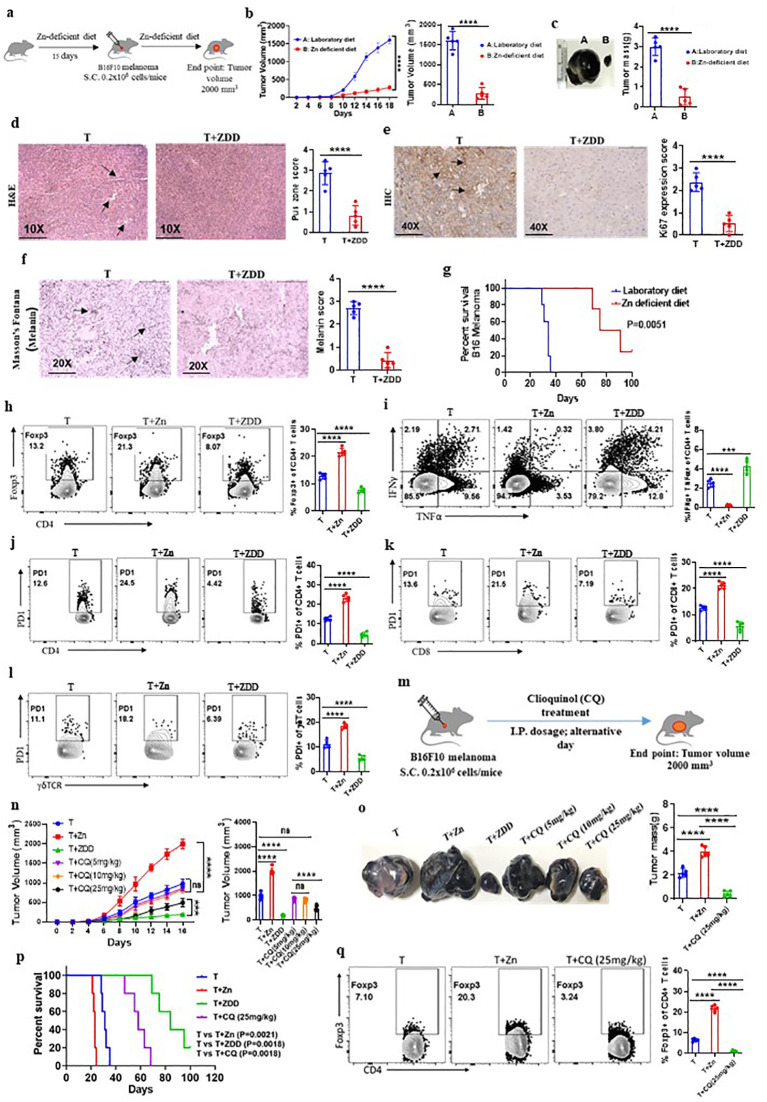
Dietary Zn deficiency and tissue Zn chelation promote antitumor immunity. **(A)** Schematic representation of the experiment in which C57BL/6 mice were fed with a Zn-deficient diet (0.85 ppm) for 15 days before B16F10 melanoma implantation and the diet was continued until the endpoint of the experiment. **(B)** Rate of growth of tumor volume in the groups of mice consuming a normal diet (T) and a Zn-deficient diet (T+ZDD). **(C)** The tumor mass in the groups of mice consuming a normal diet (T) and a Zn-deficient diet (T+ZDD). **(D–F)** Histology of the tumor tissue samples: hematoxylin and eosin (H&E) stain (×10) to identify pus zones **(D)**, immunohistochemistry (IHC) (×40) to identify Ki67 expression **(E)**, and Masson’s Fontana stain (×20) to identify the level of melanin production **(F)**. **(G)** Percent survival curve of B16F10 melanoma-bearing mice consuming a laboratory diet vs. a Zn-deficient diet. **(H)** Immunophenotyping of Foxp3^+^ Treg cells (regulatory T cells) from the tumor-infiltrated lymphocytes (TILs) of B16F10 melanoma-bearing C57BL/6 mice consuming a normal diet (T), high Zn intake (T+Zn), and a Zn-deficient diet (T+ZDD). **(I)** Immunophenotyping of IFN-γ^+^ TNF-α^+^ CD4^+^ T (Th1) cells from the TILs of T, T+Zn, and T+ZDD. **(J–L)** Immunophenotyping of PD-1^+^ CD4^+^ T cells, PD-1^+^ CD8+ T cells, and PD-1^+^ γδTCR^+^ T cells from the TILs of T, T+Zn, and T+ZDD. **(M)** Schematic representation of the experiment in which B16F10 melanoma was implanted in C57BL/6 mice and clioquinol (CQ) dose was given (i.p.) as a therapy every alternative day starting from day 2 until the tumor volume reached 2,000 mm^3^. **(N)** The rate of growth of tumor volume in the groups of mice consuming a normal diet (T), high Zn intake (T+Zn), and a Zn-deficient diet (T+ZDD) and undergoing CQ treatment of 5 mg/kg [T+CQ (5 mg/kg)], 10 mg/kg [T+CQ (10 mg/kg)], and 25 mg/kg [T+CQ (25 mg/kg)]. **(O)** The endpoint tumor mass in T, T+Zn, T+ZDD, T+CQ (5 mg/kg), T+CQ (10 mg/kg), and T+CQ (25 mg/kg). **(P)** Percent survival curve of the B16F10 melanoma-bearing group of mice consuming a normal diet (T), high Zn intake (T+Zn), and a Zn-deficient diet (T+ZDD) and undergoing CQ` treatment of 25 mg/kg [T+CQ (25 mg/kg)]. **(Q)** Immunophenotyping of Foxp3^+^ Treg cells from the TILs of B16F10 melanoma-bearing C57BL/6 mice consuming a normal diet (T) and a high Zn intake (T+Zn) and undergoing CQ treatment of 25 mg/kg [T+CQ (25 mg/kg)]. Data are representative of the mean ± SEM from three independent experiments. *P < 0.05, **P < 0.01, ***P < 0.001, and ****P < 0.0001 (Student’s *t*-test or one-way ANOVA); percent survival by the Mantel–Cox test; number of animals (*n* = 7) in all experiments.

To examine the effect of low dietary Zn on tumor immunity, we used C57BL/6 mice and fed them with a Zn-deficient diet (0.85 ppm) for 15 days before B16F10 melanoma implantation and continued the diet until the endpoint of the experiment (**Figure 3A**). The group consuming a Zn-deficient diet (T+ZDD) showed a lower growth rate of tumor volume as compared to the control group consuming a normal diet (T) (**Figure 3B**). The endpoint tumor mass was also found to be lower in the T+ZDD group as compared to the T group (**Figure 3C**). The tumor tissues were used for histology and stained with hematoxylin and eosin to measure the number of pus zones. Immunohistochemistry using the α-Ki67 antibody was used to determine the level of Ki67 expression, and Masson’s Fontana stain was used to determine the melanin production. Mice kept on a Zn-deficient diet significantly reduced the number of pus zones, Ki67 staining, and melanin, thus indicating a less aggressive tumor (**Figures 3D–F**). We performed a survival study of two groups of B16F10 melanoma-bearing mice: the group consuming a Zn-deficient diet had a higher survival rate as compared to the group that was consuming a normal diet (**Figure 3G**). We isolated lymphocytes from the spleen, lymph nodes, and tumor microenvironment from B16F10 melanoma-bearing mice consuming a normal diet (T), a high Zn diet (T+Zn), and a Zn-deficient diet (T+ZDD) and performed immunophenotyping. Our data showed that the frequency of tumor-infiltrated Foxp3^+^ Tregs was decreased in the mice that were given a Zn-deficient diet while a higher Zn intake increased the frequency of Foxp3^+^ Tregs (**Figure 3H**). In contrast, a Zn-deficient diet enhanced the frequency of tumor-infiltrated IFN-γ^+^ TNF-α^+^ CD4^+^ T cells, while the intake of high Zn reduced the frequency of these cells (**Figure 3I**). We further showed that a Zn-deficient diet reduced the PD-1 expression on tumor-infiltrating CD4^+^, CD8^+^, and γδ^+^ T cells in contrast to high Zn intake (**Figures 3J–L**). We also tested the expression of major genes that were involved in Treg differentiation and function and found that a high Zn intake increased the expression of Foxp3, TGF-β, and NFAT1 in TILs (**Supplementary Figure 12**). In contrast, mice fed with a Zn-deficient diet reduced the expression of Foxp3, TGF-β, and NFAT1 in TILs (**Supplementary Figure 12**). Moreover, the expression of Th1-specific genes such as T-bet, IFN-γ, TNF-α, and IL-2 was suppressed with a high Zn intake, while a Zn-deficient diet increased their expression in TILs (**Supplementary Figure 13**). The relative mRNA expression level of PD-1 was enhanced with high Zn intake and suppressed with dietary Zn deficiency (**Supplementary Figure 14**).

A low dietary intake of Zn or a Zn-deficient diet is not a feasible option as most of the food that we consume has Zn. Therefore, we tested whether tissue Zn chelation with Zn chelators such as CQ can be used as an effective alternative to a Zn-deficient diet for inhibiting tumor growth by enhancing antitumor immunity. To test this, we implanted B16F10 melanoma cells in C57BL/6 mice and started giving CQ doses (i.p.) as therapy every alternative day starting from day 2 (**Figure 3M**). We tested different doses of CQ to identify the suboptimal and optimal dosage of CQ that can suppress tumor growth effectively. We found that 10 mg/kg of body weight acts as the suboptimal dose of CQ (this dose of CQ is ineffective in suppressing tumor growth) while 25 mg/kg of body weight is the optimal dosage of CQ (this dose of CQ effectively suppresses tumor growth) (**Figure 3N**). Zn chelation with CQ at an optimal dose effectively suppressed tumor mass and increased the survival of the B16F10 melanoma-bearing C57BL/6 mice (**Figures 3O, P**). We isolated lymphocytes from the tumor microenvironment of tumor-bearing mice treated with or without CQ (25 mg/kg). The immunophenotyping indicated that the frequency of Foxp3^+^ Tregs was found to be decreased in the CQ-treated group as compared to the untreated group (**Figure 3Q**). Interestingly, CQ treatment did not alter the level of IFN-γ^+^ TNF-α^+^ CD4^+^ T cells (**Supplementary Figure 15A**) and PD-1^+^ T cells infiltrated into the tumor (**Supplementary Figure 15B**). To determine whether CQ-mediated tumor suppression is T-cell-dependent or not, we designed an experiment in which CQ treatment is given to RAG1^−/−^ mice. B16F10 melanoma was implanted in two groups of RAG1^−/−^ mice, of which one of the groups received CQ treatment as therapy. B16F10 melanoma-bearing C57BL/6 mice with or without CQ treatment was used as control. We found that CQ treatment was not effective in suppressing the tumor mass in RAG1^−/−^ mice; however, in C57BL/6 mice, CQ treatment effectively suppressed tumor mass (**Supplementary Figures 16A–C**). This proves that CQ-mediated tumor suppression is T-cell-dependent. Taken together, these data suggested that Zn deficiency either through diet or through chelation can enhance antitumor immunity.

### High Zn intake induces tumor progression through Treg cells

**Figure 4:**
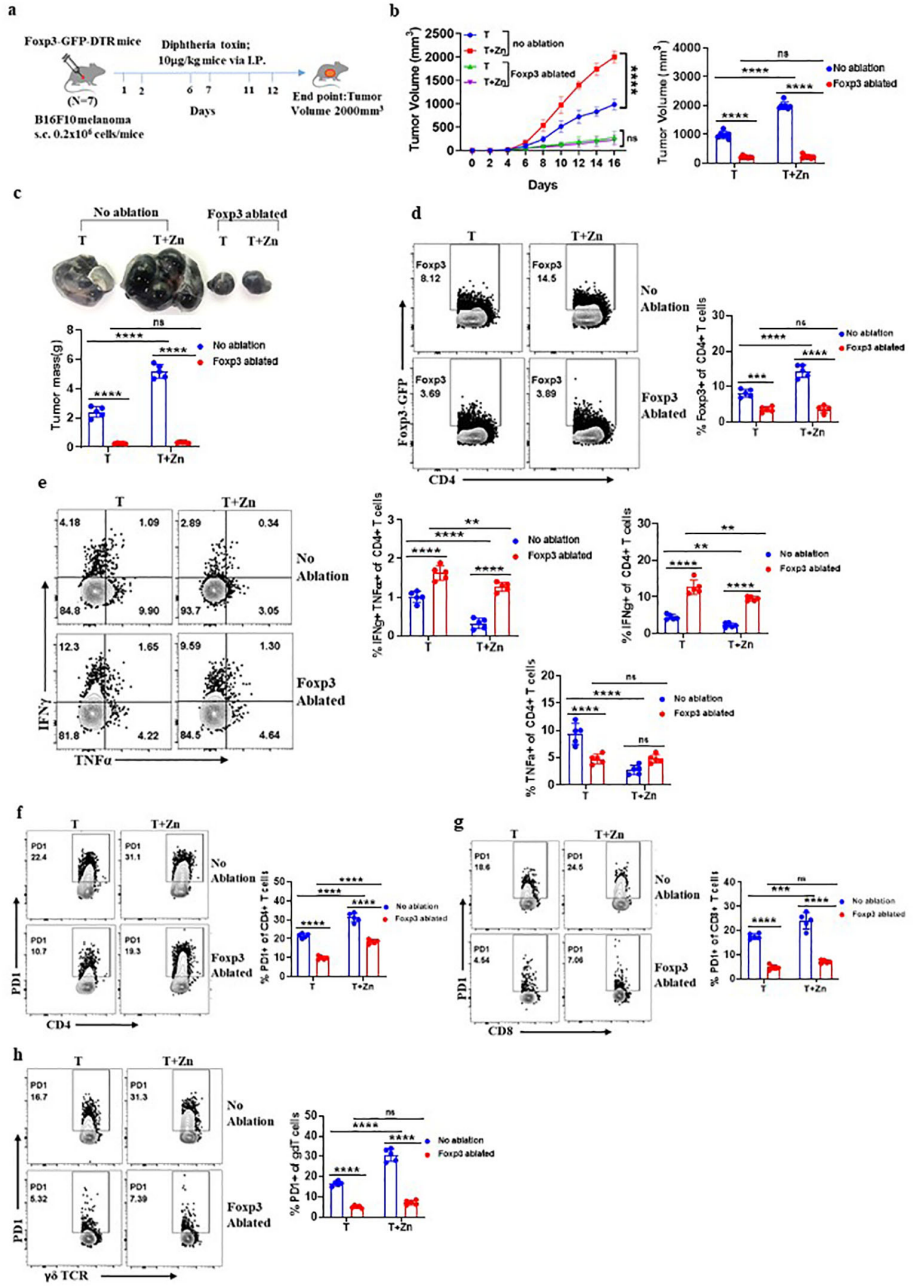
High Zn intake induces tumor progression through regulatory T cells (Tregs). **(A)** Schematic representation of the experiment in which B16F10 melanoma was implanted in FOXP3-GFP-DTR mice and these mice were given diphtheria toxin (DT) (10 μg/kg) doses for the targeted depletion of Treg cells. **(B)** The rate of growth of tumor volume in the groups of mice consuming a normal diet (T) and a high Zn intake (T+Zn), with or without Foxp3 ablation. **(C)** The tumor mass in the groups of mice consuming a normal diet (T) and a high Zn intake (T+Zn), with or without Foxp3 ablation. **(D)** Immunophenotyping of Foxp3^+^ Treg cells from the tumor-infiltrated lymphocytes (TILs) of B16F10 melanoma-bearing Foxp3-GFP-DTR mice consuming a normal diet (T) and a high Zn intake (T+Zn), with or without Foxp3 ablation. **(E)** Immunophenotyping of IFN-γ^+^ TNF-α^+^ CD4^+^ T (Th1) cells from the TILs of T and T+Zn, with or without Foxp3 ablation. **(F)** Immunophenotyping of PD-1^+^ CD4^+^ T cells from the TILs of T and T+Zn, with or without Foxp3 ablation. **(G)** Immunophenotyping of PD-1^+^ CD8^+^ T cells from the TILs of T and T+Zn, with or without Foxp3 ablation. **(H)** Immunophenotyping of PD-1^+^ γδTCR^+^ T cells from the TILs of T and T+Zn, with or without Foxp3 ablation. Data are representative of the mean ± SEM from three independent experiments. *P < 0.05, **P < 0.01, ***P < 0.001, and ****P < 0.0001 (Student’s *t*-test or one-way ANOVA); number of animals (*n* = 7) in all experiments.

Our experiments indicated that high Zn intake increases the frequency of tumor-infiltrating Foxp3^+^ Tregs while Zn deficiency or chelation reduces the frequency of tumor-infiltrating Foxp3^+^ Tregs. Moreover, high Zn intake and Zn-deficient diet/Zn chelation have opposite outcomes on tumor progression. Taken together, these observations indicated that Zn-mediated tumor progression is linked with Foxp3^+^ Tregs. To determine the role of Foxp3^+^ Tregs in Zn-mediated regulation of tumor immunity, we used FOXP3-GFP-DTR mice. In these mice, the FOXP3 gene was fused with a gene that codes for green fluorescent protein (GFP) and diphtheria toxin receptor (DTR) (38). This fusion protein allows visual identification and tracking of Tregs using flow cytometry. Additionally, these mice allow specific ablation of Tregs upon administration of diphtheria toxin (DT), as DTR is expressed under the Foxp3 gene locus. We implanted B16F10 melanoma in FOXP3-GFP-DTR mice and gave DT (10 μg/kg) doses as indicated and described earlier (39, 40) (**Figure 4A**). The tumor-alone group and the high Zn-supplemented group without DT treatment were taken as control. Our data show that the rate of tumor growth in both the DT-administered groups with or without Zn intake was found to be the same (**Figure 4B**). While high Zn intake increased the tumor growth in the control group, high Zn intake failed to increase tumor mass in DT-induced Foxp3^+^ Treg-ablated mice (**Figure 4C**). Moreover, immunophenotyping showed complete ablation of Foxp3^+^ Tregs in the DT-treated as compared to the untreated group (**Figure 4D**), confirming the depletion of Foxp3^+^ Tregs. Interestingly, tumor-infiltrating Th1 cells (IFN-γ^+^ TNF-α^+^ CD4^+^ T cells) were found to be increased in the DT-mediated Foxp3^+^ Treg-ablated group in comparison to the control (**Figure 4E**). Zn supplementation suppressed Th1 cells in the control group as shown earlier (**Figure 2E**). These data clearly showed that Zn-mediated suppression of Th1 cells is dependent on Foxp3^+^ Tregs. PD-1 expression on the tumor-infiltrated CD4 T cells, CD8 T cells, and γδT cells were suppressed in the DT-treated groups with or without Zn supplementation (**Figures 4F–H**). However, Zn supplementation in the control group increased tumor-infiltrated PD-1^+^ T cells. This confirms that PD-1 expression in T cells is influenced by Tregs.

### FOXO1 plays a role in Zn-mediated regulation of Treg cells

**Figure 5:**
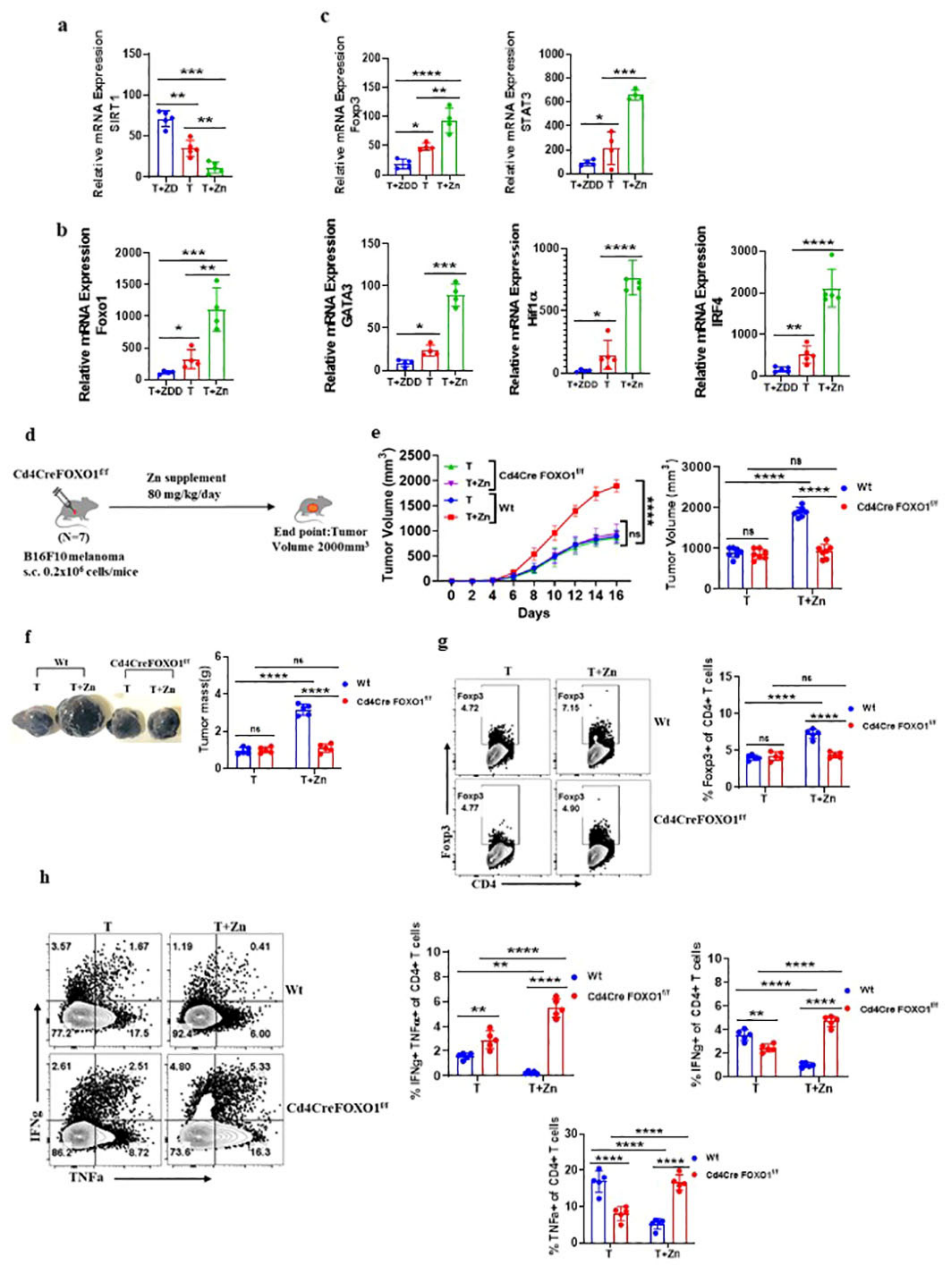
FOXO1 plays a major role in Zn-mediated regulation of Treg cells. **(A)** The relative mRNA expression level of SIRT1 in tumor-infiltrated lymphocytes (TILs) of B16F10 melanoma-bearing C57BL/6 mice consuming a normal diet (T), a Zn supplement (T+Zn), and a Zn-deficient diet (T+ZDD). **(B)** The relative mRNA expression level of the FOXO1 gene in TILs of T, T+Zn, and T+ZDD. **(C)** The relative mRNA expression level of Foxp3 and its characteristic cofactors in the TILs of T, T+Zn, and T+ZDD. **(D)** Schematic representation of the experiment in which B16F10 melanoma was implanted in CD4 conditional FOXO1-deficient (Foxo1^fl/fl^CD4Cre^+^) mice and C57BL/6 mice (wild-type, Wt). **(E)** The rate of growth of tumor volume in the groups of mice consuming a normal diet (T) and a high Zn intake (T+Zn) in the Wt group vs. the Cd4CreFOXO1^f/f^ group. **(F)** The tumor mass in the groups of mice consuming a normal diet (T) and a high Zn intake (T+Zn) in the Wt group vs. the Cd4CreFOXO1^f/f^ group. **(G)** Immunophenotyping of Foxp3^+^ Treg cells (regulatory T cells) from the TILs of the B16F10 melanoma-bearing Wt group and the Cd4CreFOXO1^f/f^ group consuming a normal diet (T) and a high Zn intake (T+Zn). **(H)** Immunophenotyping of IFN-γ^+^ TNF-α^+^ CD4^+^ T (Th1) cells from the TILs of the Wt group and the Cd4CreFOXO1^f/f^ group consuming a normal diet (T) and a high Zn intake (T+Zn). Data are representative of the mean ± SEM from three independent experiments. *P < 0.05, **P < 0.01, ***P < 0.001, and ****P < 0.0001 (Student’s *t*-test or one-way ANOVA); number of animals (*n* = 7) in all experiments.

We identified that a Zn-deficient diet induced silent information regulator 2 homolog 1 (SIRT1), enhancing the metabolic pathways (41) (**Supplementary Figures 17A–C**). SIRT1 is a protein that belongs to the family of sirtuins that modulates the activity of several transcription factors, including Forkhead box protein O1 (FOXO1). The relative mRNA expression level of SIRT1 was high in the groups with a Zn-deficient diet as compared to the Zn-supplemented groups (**Figure 5A**). The level of nicotinamide metabolite which is the by-product of SIRT1 activity was found to increase with a Zn-deficient diet and was reduced in the Zn-supplemented group (41) (**Supplementary Figure 17D**). In line with this, the relative mRNA expression of FOXO1 was found to be suppressed in TILs with a Zn-deficient diet, while the expression of FOXO1 was enhanced with Zn supplementation (**Figure 5B**). It is already known that SIRT1 suppresses FOXO1 activity through a process known as deacetylation (42). By deacetylating FOXO1, SIRT1 reduces its activity and prevents it from binding to target genes and initiating their expression (42). We also found that the relative mRNA expression level of the Foxp3 gene and its characteristic cofactors were suppressed by a Zn-deficient diet and enhanced with Zn supplementation (**Figure 5C**). It is already known that FOXO1 can directly bind to the Foxp3 gene locus and enhance its transcriptional activity (43). Thus, we are hypothesizing that Zn somehow regulates FOXO1, which stabilizes the function of Foxp3^+^ Tregs. To test our hypothesis, we implanted B16F10 melanoma in CD4 conditional FOXO1-deficient (Foxo1^fl/fl^CD4Cre^+^) mice and gave a Zn supplement as shown in **Figure 5D**. The Foxo1^fl/fl^CD4Cre^−^ mice bearing B16F10 melanoma were used as control. Foxo1^fl/fl^CD4Cre^+^ mice have the FOXO1 gene selectively deleted in CD4^+^ T cells, allowing us to investigate the T-cell-specific role of FOXO1 in tumor progression with or without a high Zn diet. The rate of growth of tumor volume was the same in both the Foxo1^fl/fl^CD4Cre^+^ mice groups with or without Zn treatment (**Figure 5E**). However, high Zn supplementation failed to increase the tumor mass in Foxo1^fl/fl^CD4Cre^+^ mice as compared to Zn-supplemented Foxo1^fl/fl^CD4Cre^−^ mice, which show an increase in tumor burden (**Figure 5F**). Immunophenotyping shows that the frequency of tumor-infiltrating Tregs was not increased in Foxo1^fl/fl^CD4Cre^+^ mice in comparison to Foxo1^fl/fl^CD4Cre^−^ mice with Zn supplementation (**Figure 5G**), suggesting that FOXO1 is essential for Zn-mediated Foxp3 Treg function in promoting tumor growth. Moreover, Zn supplementation enhanced the tumor-infiltrating Th1 cells in Foxo1^fl/fl^CD4Cre^+^ mice as compared to Foxo1^fl/fl^CD4Cre^−^ mice (**Figure 5H**). The IFN-γ^+^ CD4^+^ T cells and TNF-α^+^ CD4^+^ T cells were also increased with Zn supplementation in Foxo1^fl/fl^CD4Cre^+^ mice. This result showed that Th1 cells are enhanced in the absence of FOXO1 and Zn-mediated suppression of Th1 cells is FOXO1-dependent.

### Clioquinol enhances α-PD-1 immunotherapy in melanoma

**Figure 6:**
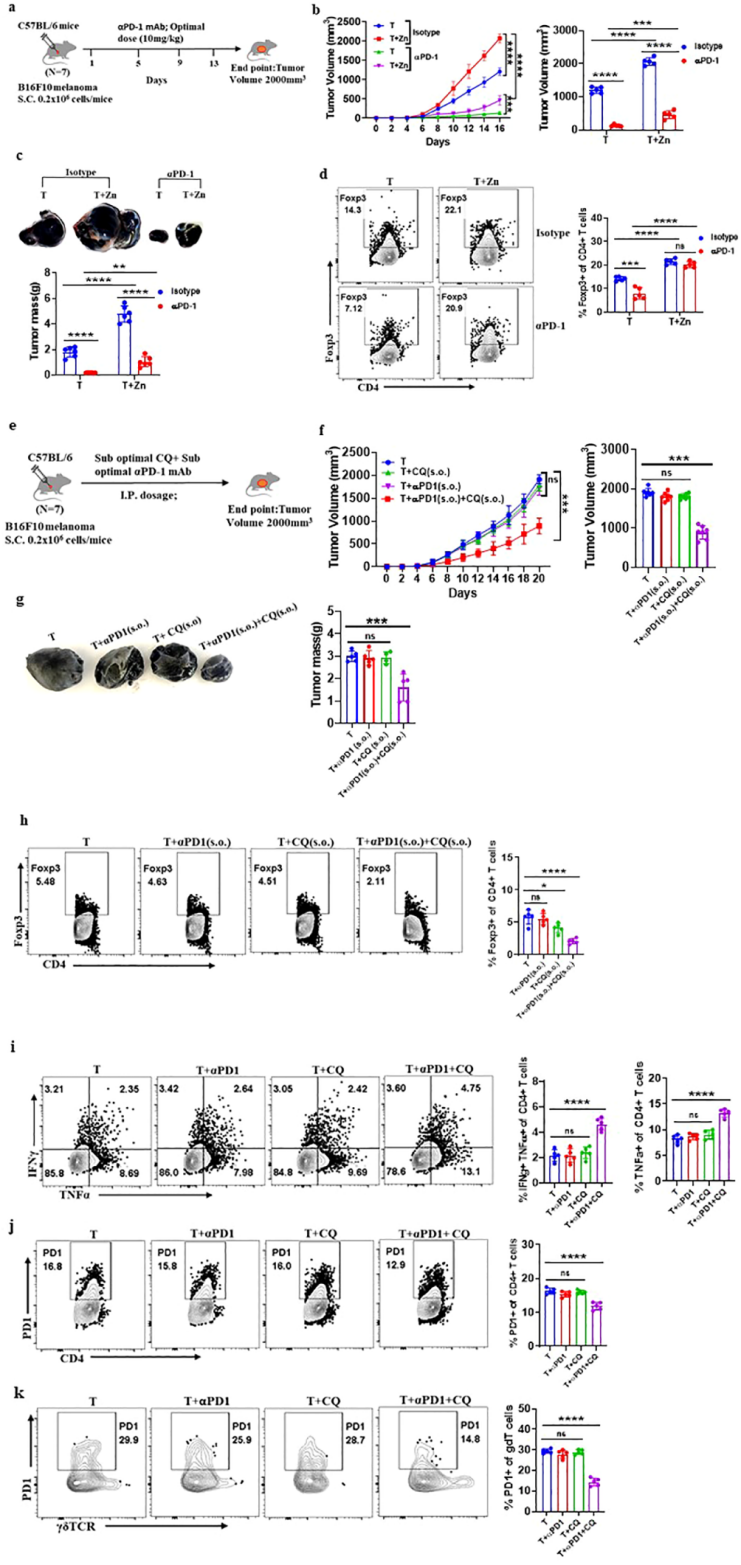
Clioquinol (CQ) can enhance α-PD-1 immunotherapy in melanoma. **(A)** Schematic representation of the experiment in which B16F10 melanoma was implanted in C57BL/6 mice of which one of the groups was given a high Zn diet while the other group was kept on a normal laboratory diet. Both groups were treated with an optimal dose of α-PD-1 mAb (10 mg/kg). **(B)** The rate of growth of tumor volume in groups of C57BL/6 mice consuming a normal diet (T) and a high Zn intake (T+Zn), which are given isotype or α-PD-1 mAb treatment. **(C)** The tumor mass in T and T+Zn, which are given isotype or α-PD-1 mAb treatment. **(D)** Immunophenotyping of Foxp3^+^ Treg cells (regulatory T cells) from the tumor-infiltrated lymphocytes (TILs) of B16F10 melanoma-bearing C57BL/6 mice consuming a normal diet (T) and a high Zn intake (T+Zn), which are given isotype or α-PD-1 mAb treatment. **(E)** Schematic representation of the experiment in which B16F10 melanoma was implanted in C57BL/6 mice, which were treated with a combination therapy of a suboptimal dose of α-PD-1 mAb (5 mg/kg) and a suboptimal dose of CQ (10 mg/kg) until the endpoint tumor volume reached 2,000 mm^3^. **(F)** The rate of growth of tumor volume in groups of C57BL/6 mice bearing B16F10 melanoma, with the tumor-only control group (T) treated with a suboptimal dose of α-PD-1 mAb (T+α-PD-1), treated with a suboptimal dose of CQ (T+CQ), and treated with a combination of a suboptimal dose of α-PD-1 mAb and a suboptimal dose of CQ (T+α-PD-1+CQ). **(G)** The tumor mass of groups T, T+α-PD-1, T+CQ, and T+α-PD-1+CQ. **(H)** Immunophenotyping of Foxp3^+^ Treg cells (regulatory T cells) from the tumor-infiltrated lymphocytes (TILs) of groups T, T+α-PD-1, T+CQ, and T+α-PD-1+CQ. **(I)** Immunophenotyping of IFN-γ^+^ TNF-α^+^ CD4^+^ T (Th1) cells from the TILs of groups T, T+α-PD-1, T+CQ, and T+α-PD-1+CQ. **(J)** Immunophenotyping of PD-1^+^ CD4^+^ T cells from the TILs of groups T, T+α-PD-1, T+CQ, and T+α-PD-1+CQ. **(K)** Immunophenotyping of PD-1^+^ γδTCR^+^ T cells from the TILs of groups T, T+α-PD-1, T+CQ, and T+α-PD-1+CQ. Data are representative of the mean ± SEM from three independent experiments. *P < 0.05, **P < 0.01, ***P < 0.001, and ****P < 0.0001 (Student’s *t*-test or one-way ANOVA); number of animals (*n* = 7) in all experiments.

To investigate the role of Zn in determining the success of α-PD-1 checkpoint therapy, we designed an experiment in which an optimal dose of α-PD-1 monoclonal antibody (10 mg/kg) was used as a therapeutic approach. We implanted B16F10 melanoma in C57BL/6 mice of which one of the groups was given a high Zn diet while the other group was kept on a normal laboratory diet. Both groups were treated with an optimal dose of α-PD-1 monoclonal antibody (10 mg/kg) as shown in **Figure 6A**. The tumor-bearing C57BL/6 mice consuming a high Zn diet and a normal diet without α-PD-1 antibody treatment were used as control. α-PD-1 treatment suppressed tumor growth in the treatment-only group. The rate of tumor growth was lower in the α-PD-1 antibody-treated group consuming a normal diet as compared to the group with high dietary Zn intake (**Figure 6B**). The α-PD-1 monoclonal antibody therapy effectively suppressed tumor mass in the treatment-only group. However, the treatment group which was receiving a high Zn intake along with the α-PD-1 antibody did not respond well to the therapy (**Figure 6C**). The immunophenotyping of tumor-infiltrated immune cells showed higher levels of Foxp3^+^ Treg in the α-PD-1 antibody-treated group consuming high Zn as compared to the treatment-only group (**Figure 6D**). An increase in tumor-infiltrated Foxp3^+^ Tregs was also found in the untreated control group consuming a high dietary Zn. Although there was an increase in the level of tumor-infiltrated Th1 cells in the α-PD-1 antibody-treated groups, the inclusion or exclusion of the Zn supplement did not cause any difference (**Supplementary Figure 18A**). The IFN-γ^+^ CD4^+^ T cells and TNF-α^+^ CD4^+^ T cells also did not show any changes with high Zn intake in the α-PD-1-treated groups (**Supplementary Figure 18A**). However, in the untreated control group, Th1 cells were suppressed with high Zn intake.

α-PD-1 checkpoint therapy resulted in the reduction of PD-1^+^ T cells infiltrating the tumor microenvironment as compared to the untreated control groups. However, PD-1 expression on tumor-infiltrated T cells was high in the α-PD-1 antibody-treated group with high Zn intake as compared to the treatment-only group (**Supplementary Figures 18B–D**). High Zn intake in the untreated control group also increased PD-1 expression in T cells. This shows that α-PD-1 therapy will not be effective with the intake of high dietary Zn. We wanted to determine if Zn chelators can be used as an effective therapy with α-PD-1 checkpoint inhibitors.

As we have already shown that the tumor-bearing mice with high Zn intake responded low to α-PD-1 checkpoint therapy, we wanted to know if the use of tissue Zn chelators as a combination therapy with α-PD-1 checkpoint inhibitors works effectively. We used CQ as the tissue Zn chelator because it removes only free chelatable Zn pools and does not remove Zn associated with proteins (44). We have shown that 25 mg/kg of CQ is the optimal dose and effectively suppresses tumor mass while 10 mg/kg is the suboptimal dose which independently is not effective. It is already known in earlier studies that a suboptimal dose of α-PD-1 monoclonal antibody (5 mg/kg) as a treatment independently is not effective in suppressing tumor (16). We implanted B16F10 melanoma in C57BL/6 mice and were treated with a combination of a suboptimal dose of Zn chelator and a suboptimal dose of α-PD-1 monoclonal antibody as shown in **Figure 6E**. Each of these therapies independently was not effective in suppressing tumors; however, the rate of growth of tumor volume was suppressed considerably with the combination therapy (**Figure 6F**). The combination therapy successfully suppressed the tumor mass (**Figure 6G**). The immunophenotyping of tumor-infiltrated immune cells showed lower levels of Foxp3^+^ Tregs in the group that received combination therapy compared with the groups that received each of these therapies independently (**Figure 6H**). The level of tumor-infiltrated Th1 cells was high in the group that received combination therapy (**Figure 6I**). TNF-α^+^ CD4^+^ T cells were enhanced with the combination therapy; however, there was no change in the level of IFN-γ^+^ CD4^+^ T cells. The combination therapy also suppressed the PD-1 expression on CD4 T cells and γδ T cells; however, the individual therapy did not cause any change (**Figures 6J, K**). This result showed that the Zn chelator CQ can be combined with α-PD-1 monoclonal antibody for effective therapeutic outcomes.

## Discussion

Cancer is the leading cause of death worldwide, accounting for an estimated 9.9 million deaths in 2020 (45). Between 2010 and 2019, the global cancer incidence increased by 26%, alongside a 21% increase in cancer deaths (46). This demands a betterment in the current treatment regimes. In the current study, we have tried to add another dimension to cancer treatment by unraveling the role of Zn in tumor immune modulation. Many studies have reported that long-term intake of high Zn supplement increases the risk of prostate cancer (19, 47, 48). *In-vitro* Zn supplementation to the mixed lymphocyte cultures results in increased levels of regulatory T cells (49–51). In most of the tumors, high levels of serum Zn concentration and tissue Zn concentration are recorded (52–55). However, there is no study available on the precise role of Zn on tumor development. In the current study, we investigated the role of dietary zinc on tumor progression and antitumor immunity.

Zn is commercially available in the form of supplements that contain 7–80 mg of elemental Zn (56). Multivitamins are used by 62% of the adult population and have 7.5–15 mg of elemental Zn (56). The recommended daily intake of Zn for adults is 8–11 mg (57). Research shows that long-term usage of high doses of Zn supplements has adverse effects such as suppressed immune system, copper deficiency, genitourinary complications, anemia, and decreased high-density lipoprotein cholesterol levels (58). As Zn supplementation is widely used, a detailed study analyzing the effects of Zn on tumor progression has become vital. Some research says that Zn is important for tumor growth as dietary Zn deficiency is known to inhibit several types of animal tumors (59–62). As cells turn cancerous, they develop certain mechanisms to shutdown Zn efflux, thus maintaining intracellular Zn concentrations (63). The rapidly dividing cells require augmented cellular Zn uptake as per the results of studies of zinc transporter silencing (64, 65). In our study, we have identified that intake of high dietary Zn for longer duration during tumor development results in increased tumor growth and increased tumor metastasis. For our study, we used a solution of zinc sulfate heptahydrate dissolved in distilled water so that each animal received 2.5 mg of zinc daily corresponding to approximately 80 mg/kg. We have not encountered any symptoms of toxicity in our animal model as many studies used similar levels of zinc in their studies (66–68). The post-surgical tumor recurrence also increased with high dietary Zn. The overall survival rate is decreased with increased intake of Zn. Many studies have reported that *in-vitro* Zn supplementation to the mixed lymphocyte cultures results in increased levels of Treg cells (49, 50). We showed that the Zn-mediated effect on tumor immune response mainly comes through the T-cell compartment. Zn exerts its effect on the immune system primarily through Th (T helper) lymphocytes (23). We further went on to identify that Zn supplementation during tumor development alters mainly CD4 T-cell subtypes by suppressing the T helper 1 (Th1) cell population and enhancing the Foxp3^+^ Treg cell population. Zn plays an important role in proinflammatory cytokine regulation as Zn deficiency induces the production of proinflammatory cytokines (69). This strengthens our finding that elevated Zn intake suppresses IFN-γ and TNF-α in tumor-bearing mice. We showed that Zn supplementation significantly increased PD-1 immune checkpoints on CD4 T cells, CD8 T cells, and γδT cells. There are no studies available on the effect of Zn on immune checkpoints; thus, our findings open the avenue for novel therapeutic approaches. We find that the degranulation marker CD107a or LAMP1 expression on NK cells and CD8 T cells decreased by Zn supplementation indicating the decrease in cytotoxicity response among these cells. This finding is strengthened by the earlier research that the administration of high doses of Zn in mice decreases the cytotoxicity of NK cells (70). Dietary Zn depletion is known to retard tumor growth even after the tumor is very well established (60). We show that administration of a Zn-deficient diet suppresses tumor burden and increases Th1 cells while suppressing Foxp3^+^ Treg cells. Dietary Zn depletion also suppressed PD-1 expression on T cells and increased CD107a expression on NK cells and CD8 T cells leading to high cellular cytotoxicity among these cells. Zn supplementation increases the frequency of Treg cells in *in-vitro* cultures (49, 71). Thus, to determine the role of regulatory T cells, we depleted Treg cells in a targeted manner, which alleviated the Zn-mediated effect confirming their major role. This confirms the role of Treg cells in Zn-mediated increase in tumor burden. Then, we further went into elucidating the molecular mechanism of immune modulation mediated by Zn in tumor immune response. We found that high dietary Zn increases the relative mRNA expression of FOXO1 while tumor suppression by the Zn-deficient diet results in a decrease in the level of FOXO1 mRNA expression. In general, FOXO1 is essential for the development and function of Treg cells (43); thus, we used Cd4CreFOXO1^f/f^ mice to determine the role of FOXO1 in Zn-mediated induction of Treg cells. We found that Zn intake could not increase tumor burden in FOXO1-deficient mice, and thus, we confirmed that FOXO1 plays a major role in Zn-mediated Treg cell differentiation and function. We have also identified that high Zn intake results in a lower response for α-PD-1 tumor immune checkpoint therapy. The use of tissue Zn chelators such as CQ as a combination therapy with α-PD-1 monoclonal antibody effectively suppressed tumor mass. In summary, we have shown for the first time that Zn intake negatively regulates antitumor immune response by FOXO1-mediated regulation of Treg cell differentiation and function and also suppresses α-PD-1 tumor immune checkpoint therapy response. The identification helps in designing targeted immune therapies for cancer by bringing trace element analysis to the forefront of immunotherapy and improving the outcome of immune checkpoint therapy response.

The use of Zn chelators along with a suboptimal dose of α-PD-1 mAb immune checkpoint therapy will help to overcome the drawbacks and improve the effectiveness of immune therapy for cancer. α-PD-1 mAb treatment comes with a set of drawbacks such as non-responsiveness and gastrointestinal toxicity, namely, colitis and diarrhea, and other gastrointestinal adverse effects such as decreased appetite, nausea, vomiting, and constipation (72). α-PD-1 mAb as an immune checkpoint inhibitor has shown only marginal success (73). Our study strengthens the need for complete trace element analysis of body fluids and tissues of responders vs. non-responders of α-PD-1 therapy. Since an imbalance in the level of trace elements is associated with the development of various cancers (10, 74) and also decides the therapeutic outcome (75), trace element analysis can become an essential wing of cancer immunotherapy.

Our findings open excellent therapeutic avenues for targeting cancer. Firstly, Zn chelators can be used as a tumor therapeutic agent. The Zn chelator CQ can be repurposed for cancer immunotherapy. A suboptimal dose of CQ can be combined with suboptimal α-PD-1 mAb checkpoint therapy to overcome the drawbacks of immune checkpoint therapy and for efficient treatment outcome. Secondly, trace element analysis can become the forefront of cancer immunotherapy, as our study along with many other reports proves that the level of trace elements determines tumor development and therapeutic outcome.

## Materials and methods

### Mice

C57BL/6, RAG1^−/−^, and Foxp3-GFP-DTR mice were obtained from Jackson Laboratory, USA. All the mice were housed and maintained in a conventional pathogen-free environment at the Small Animal Facility (SAF) located at the Translational Health Science and Technology Institute (THSTI). To perform the animal experiments, prior approval was obtained from the Institutional Animal Ethics Committee (IAEC project protocol approval no. IAEC/THSTI/114). All the animal experiments were handled by experienced personnel. The experiments were planned and executed according to the guidelines laid out by the Institutional Animal Ethics Committee of THSTI.

### Cell lines

B16F10 and B16F10-ova melanoma cell lines were obtained from the American Type Culture Collection (ATCC). The cell lines were maintained in R10 medium which comprises RPMI 1640 (by Invitrogen, Carlsbad, CA, USA) complete media containing 10% (v/v) fetal bovine serum (FBS) by Gibco, Waltham, MA, USA, penicillin (100 U/ml) by Sigma-Aldrich, St. Louis, MO, USA, streptomycin (100 μg/ml) by Sigma-Aldrich, St. Louis, MO, USA, L-glutamine (2 mM) by Sigma-Aldrich, St. Louis, MO, USA, and HEPES (working: 10 mM; stock: 0.5 M) by Sigma-Aldrich, St. Louis, MO, USA. In this study, we did not use any cell lines found in the Register of Misidentified Cell Lines which is maintained by the International Cell Line Authentication Committee (http://iclac.org/databases/cross-contaminations/).

### Tumor implantation

B16F10 and/or B16F10-ova cell lines were cultured in a sterile R10 medium at 37°C in an incubator maintained with 5% CO_2_. Six- to 8-week-old male mice were used in the study. Each mouse was injected with 0.2 × 10^6^ cells subcutaneously on the left flank region or injected intravenously, particularly for the metastasis studies. The mice were kept on a normal laboratory diet, a high Zn supplement, or a Zn-deficient diet as specified in each experiment. Tumor volume was recorded each day until it reached 2,000 mm^3^. The formula to measure tumor volume (mm^3^) = *L* × *W* × *W* × 0.5, where *L* = length and *W* = width of the tumor mass (in millimeters). Daily intake of food, water, and body weight was measured. Once the tumor volume reached 2,000 mm^3^, the animals were euthanized and their organs and other samples were collected to be used for various studies. For the survival studies, the animals were kept under observation, and the day of mortality of individual animals was recorded to prepare the percent survival curve for 100 days.

### Parameters evaluated to rule out toxicity by the Zn treatment

To thoroughly evaluate the potential toxicity of the Zn supplement, the following parameters were assessed. One group of healthy C57BL/6 mice was given Zn supplement (WT+Zn), while the other group was kept on a normal laboratory diet (WT):

- Clinical observations: We monitored the mice for any signs of distress, changes in behavior, or physical abnormalities.
- Body weight: We measured the body weight of the mice regularly to detect any abnormal weight changes (**Supplementary Figure 1**).
- Histopathological examination: We conducted a histological examination of major organs to assess for any morphological changes. H&E staining at ×10 of major organs including the lungs, heart, liver, and kidney was conducted for both C57BL/6 mice consuming a normal diet vs. C57BL/6 mice consuming Zn supplement (**Supplementary Figure 2**).

### Antibodies

The following antimouse antibodies were used: α-CD45.2 (1:700; 104, BioLegend, San Diego, CA, USA), α-CD3 (1:700; 145-2C11, BioLegend, San Diego, CA, USA), α-CD4 (1:1,000; GK1.5, BioLegend, San Diego, CA, USA), α-CD8 (1:1,000; 53-6.7, BioLegend, San Diego, CA, USA), α-NK1.1 (1:700; PK136, BioLegend, San Diego, CA, USA), α-γδTCR (1:700; GL3, BioLegend, San Diego, CA, USA), α-PD-1 (1:500; 29F.1A12, BioLegend, San Diego, CA, USA), α-CTLA-4 (1:500; UC10-4B9, BioLegend, San Diego, CA, USA), α-TIM3 (1:600; B8.2C12, BioLegend, San Diego, CA, USA), α-CD107a (1:700; 1D4B, BioLegend, San Diego, CA, USA), α-CD96 (1:700; 3.3, BioLegend, San Diego, CA, USA), α-TIGIT (1:700; 1G9, BioLegend, San Diego, CA, USA), α-Gr1 (1:1,500; RB6-8C5, BioLegend, San Diego, CA, USA), α-CD11b (1:1,500; M1/70, BioLegend, San Diego, CA, USA), α-F4/80 (1:700; BM8, BioLegend, San Diego, CA, USA), α-CD206 (1:700; C068C2, BioLegend, San Diego, CA, USA), α-IFN (1:500; XMG1.2, BioLegend, San Diego, CA, USA), α-TNF (1:500; MP6-XT22, BioLegend, San Diego, CA, USA), α-IL-17A (1:500; TC11-18H10, BioLegend, San Diego, CA, USA), α-perforin (1:500; S16009A, BioLegend, San Diego, CA, USA), and α-Ki67 (1:1,000; SolA15, Thermo Fisher Scientific, Waltham, MA, USA).

### Flow cytometry and intracellular cytokine staining

The organs (spleen, lymph nodes, and tumor) were processed and tissue-infiltrated lymphocytes were isolated and stained for surface markers by fluorescence-tagged antibodies. Staining for surface markers was done in fluorescence-activated cell sorting (FACS) buffer (1× PBS and 2% FBS). For intracellular cytokine staining, the cells were stimulated for cytokine production with phorbol 12-myristate13-acetate (PMA; 50 ng/ml; Sigma-Aldrich, St. Louis, MO, USA), ionomycin (1 g/ml; Sigma-Aldrich, St. Louis, MO, USA), and monensin (#554724 GolgiStop, BD Biosciences, Franklin Lakes, NJ, USA) for 4 h at 37°C with 5% CO_2_. Surface staining (1^0^ staining) was performed with antibodies diluted in FACS buffer as indicated above and incubated for 30 min at 4°C in the dark. Then, the cells were fixed in Cytofix and permeabilized with Perm/Wash buffer using the Fixation Permeabilization Solution kit (#554714, BD Biosciences, Franklin Lakes, NJ, USA). The secondary staining was then performed to stain the intracellular cytokines by diluting the antibodies in the permeabilization buffer and incubating for 30 min at 4°C in the dark. The cells were then washed and proceeded for flow cytometry (Canto II, BD Biosciences, Franklin Lakes, NJ, USA). We consistently used one million cells for each sample to ensure the reliability and reproducibility of our results. Data analysis was performed using the FlowJo software (TreeStar, Bend, OR, USA).

### Quantitative polymerase chain reaction

The total RNA was extracted from the samples using an RNeasy kit (#74104, Qiagen, Germantown, MD, USA), and complementary DNA (cDNA) was synthesized using an iScript cDNA Synthesis kit (#1708891, Bio-Rad, Hercules, CA, USA) according to the manufacturer’s instructions. Real-time qPCR was performed using the SYBR green gene expression assay and the Fast 7500 Dx qPCR System (Applied Biosystems, Waltham, MA, USA) using the following parameters: 95°C for 15 min, 40 cycles of 94°C for 15 s, 58°C for 30 s, and 72°C for 30 s. The Ct values for the individual samples (genes) were then normalized to the endogenous control GAPDH (β-actin/glyceraldehyde-3-phosphate dehydrogenase) gene expression. To determine the Ct value change (ΔCt), the Ct value of the endogenous control gene was subtracted from the Ct value of each target gene. Then, the fold change was calculated to determine the relative expression of each gene using the formula [POWER (2, −ΔCt) × 10,000] (76). All the primer sets were purchased from Sigma-Aldrich, St. Louis, MO, USA. The following primer sets were used: IFN-γ, TNF-α, T-bet, IL2, TGF-β, Foxp3, NFAT1, PD-1, FOXO1, SIRT1, and GAPDH. The results were analyzed using the SDS 2.1 software.

### Statistical analysis

Statistical analysis was carried out with GraphPad Prism 7.0 software. FACS and qPCR data were compared and analyzed using one-way ANOVA or Student’s *t*-test for *n* = 7 mice per group. Graphs were depicted as means with SEM. A *p*-value of less than 0.05 was considered statistically significant.

### Lung metastasis

B16F10 cell lines were cultured in a sterile R10 medium at 37°C in an incubator maintained with 5% CO_2_. Six- to 8-week-old male mice were injected with 0.2 × 10^6^ cells intravenously into the tail vein. The mice were then kept on a normal laboratory diet, a high Zn supplement, or a Zn-deficient diet. The lungs were isolated from the mice at different time points starting from day 15. The number of foci was counted and graphically represented. The metastasis mice were also monitored for over 100 days for the survival study where the mortality of individual mice was recorded and represented graphically.

### Histology

The tissues used for histology were fixed in 10% (v/v) formalin until the samples were sent for analysis. The tissue samples were processed and stained with hematoxylin and eosin to measure the number of pus zones. Masson’s Fontana stain was used to determine the melanin production, and immunohistochemistry using the α-Ki67 antibody was used to determine the level of Ki67 expression (77). A certified histologist through blind sampling carried out the assessment of the stained slides and gave the histological score on a scale of 0 to 5 (where 0 meant no staining).

### Isolation of tumor-infiltrating immune cells

At the end of each study, the tumors were excised and the tumor-infiltrated immune cells were isolated using the Percoll density gradient method (78). As the first step, the tumor was chopped into smaller pieces and mechanically dissociated in the presence of complete IMDM media in a C-tube using a magnetic cell sorting dissociator (Miltenyi Biotec, Auburn, CA, USA). The cells were pelleted by centrifugation at 2,000 rpm for 5 min. The pellet was then subjected to enzymatic dissociation in HBSS media or complete IMDM media that contained collagenase type IV (100 U/ml), deoxyribonuclease I (100 g/ml), and CaCl_2_/MgCl_2_ (2.5 mg/ml) (stock: 50 mg/ml) to make up the volume to 3 ml and then incubated for 30 min at 37°C in a shaker incubator. After the incubation, the dissociated tissue was passed through a 70-μm cell strainer and centrifuged at 2,000 rpm for 5 min at room temperature. Five milliliters of 63% Percoll was added to the pellet and slowly layered it with 3 ml of 47% Percoll and finally layered on the top with 2 ml of 33% Percoll. The tubes were centrifuged at 2,000 rpm for 30 min at 4°C without deacceleration. After centrifugation, the tumor-infiltrated lymphocytes obtained as a faint layer just above the 63% gradient were carefully isolated and suspended in complete IMDM media and used for further analysis.

### Immune checkpoint inhibition by anti-PD-1 therapy

Anti-PD-1 monoclonal antibody (RMP1-14, BioXCell, Lebanon, NH, USA) was used for immune checkpoint inhibition therapy. We used both suboptimal (5 mg/kg) and optimal (10 mg/kg) doses of α-PD-1 mAb for therapeutic purposes. Doses were given intraperitoneally, two times a week starting from the day when the tumor became palpable (79, 80). Mice given α-PD-1 mAb doses were kept either on a normal laboratory diet, a Zn supplement, or a Zn-deficient diet.

### Zn chelation using clioquinol

Clioquinol (Cat. No. 33931, Sigma) is a tissue Zn chelator in mice. It removes only chelatable Zn pools and not the Zn that is bound to proteins. CQ doses were given intraperitoneally as oral administration will not have any effect. Once injected, CQ is absorbed rapidly and metabolized and will cause Zn chelation within 2 h. We have used 25 mg/kg as the optimal dose and 10 mg/kg as the suboptimal dose, injected as a therapeutic dose once the tumor becomes palpable and also from the day of tumor implantation till the endpoint.

### Sorting of CD4^+^ T cells, CD8^+^ T cells, and NK cells

Sorting of CD4^+^ T cells, CD8^+^ T cells, and NK cells from isolated tumor-infiltrating immune cells was carried out on FACS Aria III (BD Biosciences, Franklin Lakes, NJ, USA) based on the surface markers CD3^+^, NK1.1^+^, CD4^+^, CD62L^+^, and CD8^+^ according to the gating strategy. The purity of the sorted cells was ~85%–95% in the post-sort analysis. Sorted cells were then *in-vitro*-activated and differentiated in the presence and absence of Zn to do further analysis. The cells were used for staining followed by FACS and also for qPCR analysis.

### *In-vitro* stimulation

For *in-vitro* activation and stimulation of freshly sorted cells, the 96-well plates were initially coated with α-CD3/α-CD28 (2 μg/ml) and incubated overnight at 4°C. On the next day, the plates were centrifuged and the supernatant was aspirated. Then, the cells were seeded (0.1 × 10^6^ cells per well) in the 96-well plate along with the respective cytokines in IMDM media and incubated for 48–72 h at 37°C in an incubator maintained with 5% CO_2_. Naive CD4 T cells were differentiated into Th1 cells using IL-12 (15 ng/ml), Th2 cells using IL-4 (30 ng/ml), Th9 cells using TGF-β (2.5 ng/ml) and IL-4 (30 ng/ml), Th17 cells using TGF-β (2.5 ng/ml), and IL-6 (25 ng/ml) and iTreg cells using TGF-β (2.5 ng/ml). Activation of NK cells was done by culturing the cells in the media containing IL-2 (1,000 U) and IL-12 (10 ng/ml) for 48–72 h (81–83). To determine the effect of Zn, we carried out the activation and differentiation of the cells with or without 50 μM of Zn supplement (ZnSO4.7H_2_O, Sigma).

## Data Availability

The original contributions presented in the study are included in the article/**Supplementary Material**. Further inquiries can be directed to the corresponding author.
