# Supplementary material for: Zinc dampens antitumor immunity by promoting Foxp3+ regulatory T cells
Source: Front Immunol. 2024 Aug 23;15:1389387. doi: 10.3389/fimmu.2024.1389387 (PMC11377231; doi:10.3389/fimmu.2024.1389387)
Supplement: Supplementary file 1 [file Presentation1.pdf]

Fig S1

a

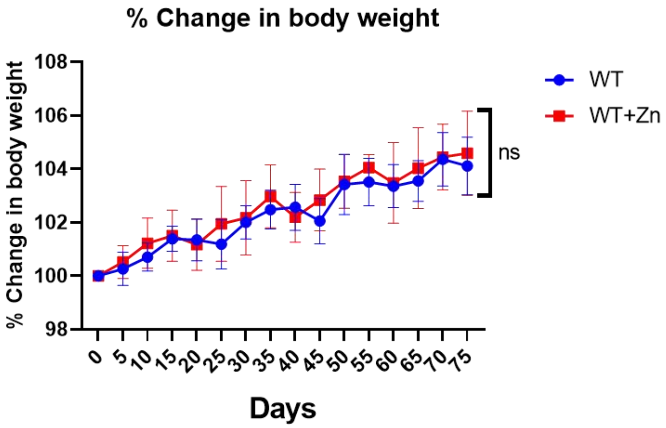

Fig S2

a

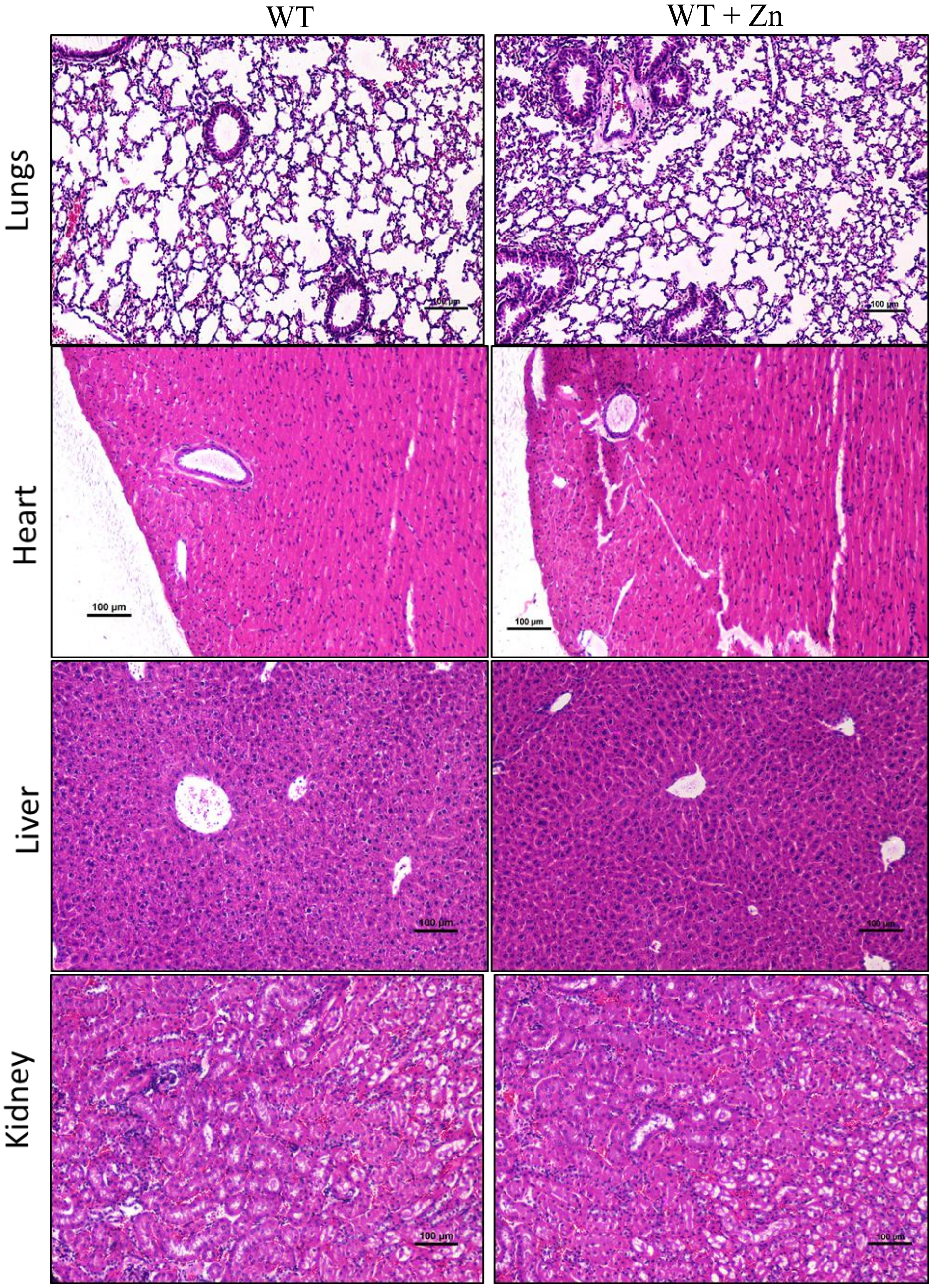

Fig. S3

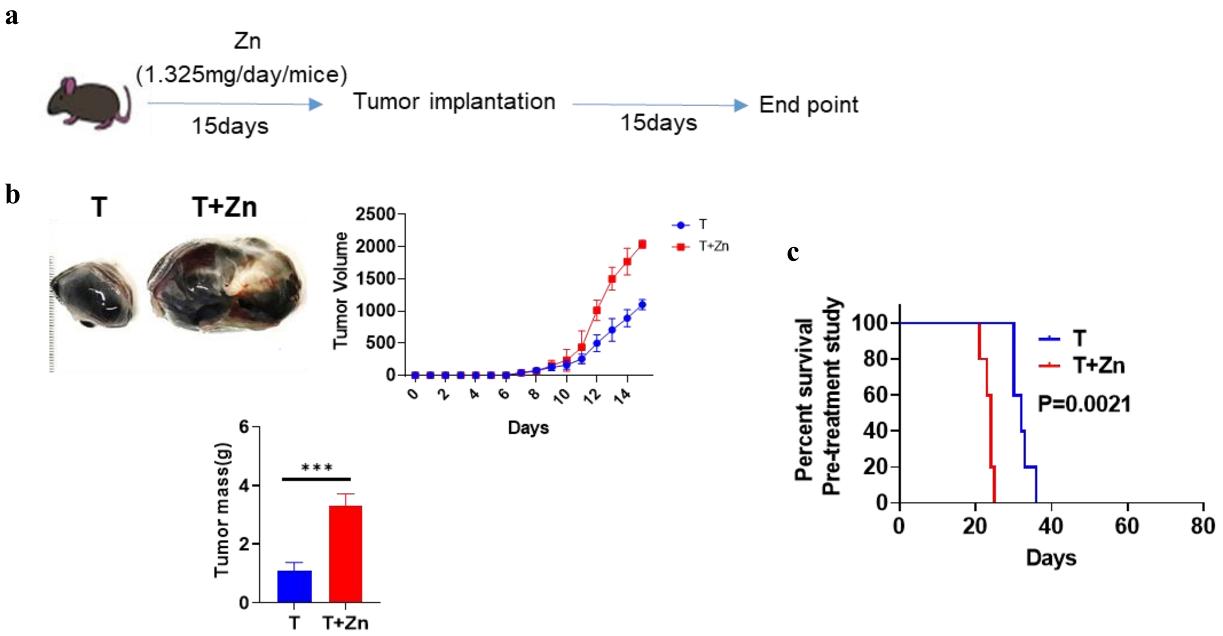
a
Zn
(1.325mg/day/mice)
15days
Tumor implantation
15days
End point
b
T
T+Zn
Tumor Volume
2500
2000
1500
1000
500
0
0
2
4
6
8
10
12
14
Days
T
T+Zn
Tumor mass(g)
6
4
2
0
***
T
T+Zn
c
Percent survival
Pre-treatment study
100
80
60
40
20
0
T
T+Zn
P=0.0021
0
20
40
60
80
Days

Fig. S4

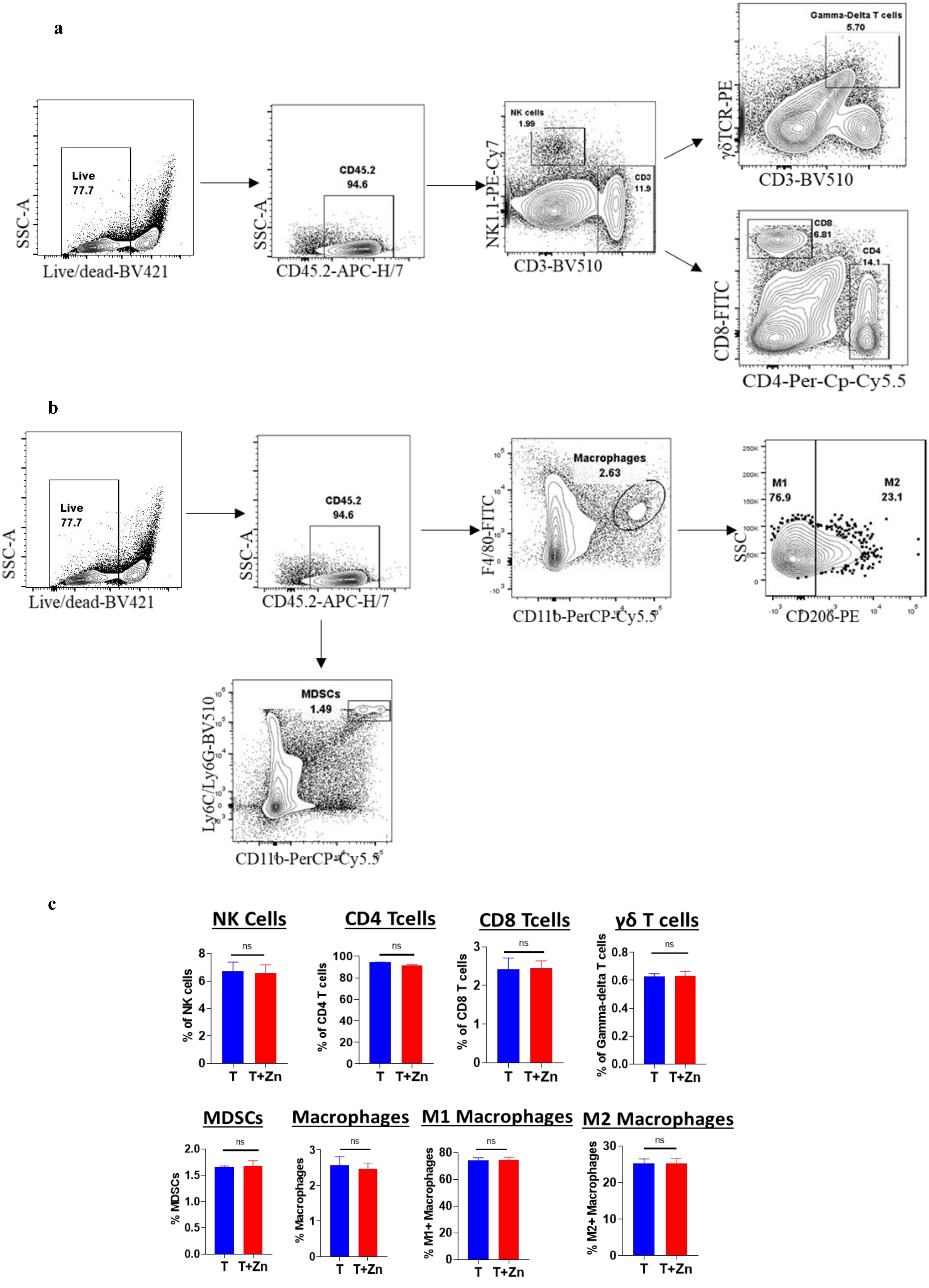
a
SSC-A
Live
77.7
Live/dead-BV421
SSC-A
CD45.2
94.6
CD45.2-APC-H/7
NK1.1-PE-Cy7
NK cells
1.99
CD3
11.9
CD3-BV510
γδTCR-PE
Gamma-Delta T cells
5.70
CD3-BV510
CD8-FITC
CD8
6.81
CD4
14.1
CD4-Per-Cp-Cy5.5
b
SSC-A
Live
77.7
Live/dead-BV421
SSC-A
CD45.2
94.6
CD45.2-APC-H/7
F4/80-FITC
Macrophages
2.63
CD11b-PerCP-Cy5.5
SSC
M1
76.9
M2
23.1
CD206-PE
Ly6C/Ly6G-BV510
MDSCs
1.49
CD11b-PerCP-Cy5.5
c
NK Cells
% of NK cells
CD4 Tcells
% of CD4 T cells
CD8 Tcells
% of CD8 T cells
γδ T cells
% of Gamma-delta T cells
MDSCs
% MDSCs
Macrophages
% Macrophages
M1 Macrophages
% M1+ Macrophages
M2 Macrophages
% M2+ Macrophages
ns
T
T+Zn

Fig. S5

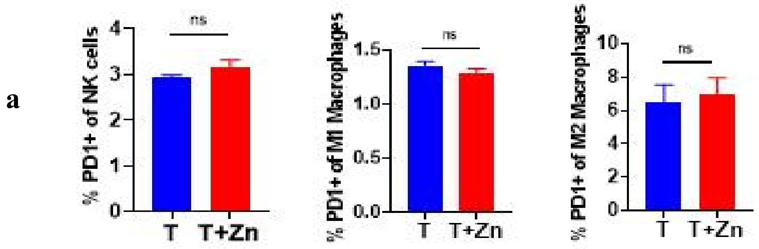
a
% PD1+ of NK cells
ns
T
T+Zn
% PD1+ of M1 Macrophages
ns
T
T+Zn
% PD1+ of M2 Macrophages
ns
T
T+Zn

Fig. S6

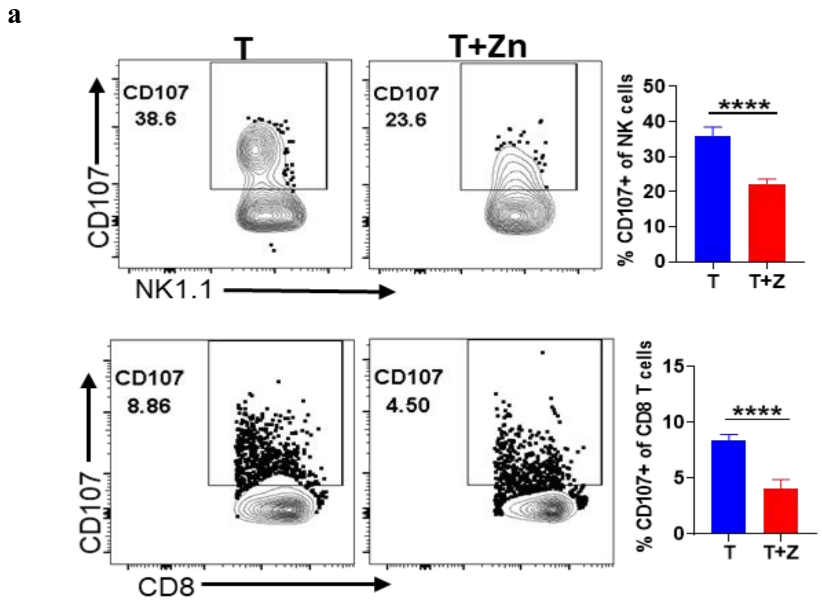
a
T
T+Zn
CD107
38.6
CD107
23.6
CD107
NK1.1
% CD107+ of NK cells
****
T
T+Z
CD107
8.86
CD107
4.50
CD107
CD8
% CD107+ of CD8 T cells
****
T
T+Z

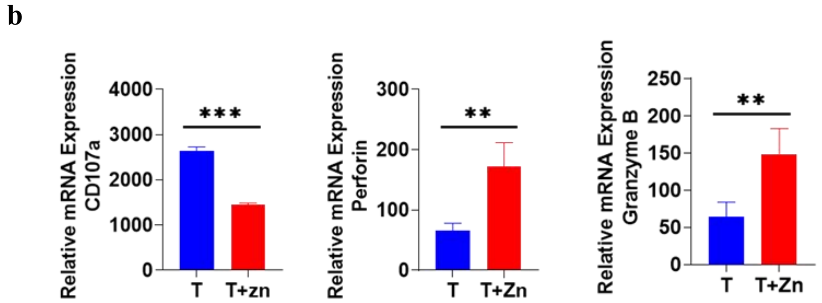
b
Relative mRNA Expression
CD107a
***
T
T+zn
Relative mRNA Expression
Perforin
**
T
T+Zn
Relative mRNA Expression
Granzyme B
**
T
T+Zn

Fig. S7

a

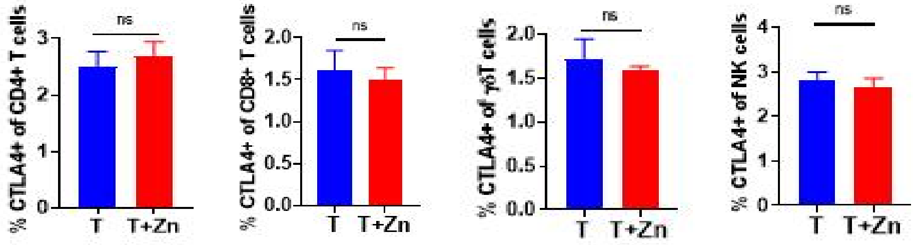
% CTLA4+ of CD4+ T cells
ns
0
1
2
3
T
T+Zn
% CTLA4+ of CD8+ T cells
ns
0.0
0.5
1.0
1.5
2.0
T
T+Zn
% CTLA4+ of γδT cells
ns
0.0
0.5
1.0
1.5
2.0
T
T+Zn
% CTLA4+ of NK cells
ns
0
1
2
3
4
T
T+Zn

b

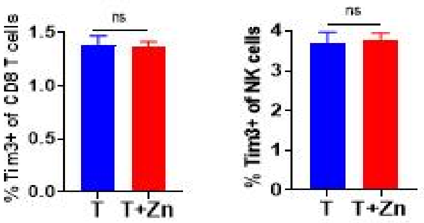
% Tim3+ of CD8 T cells
ns
0.0
0.5
1.0
1.5
T
T+Zn
% Tim3+ of NK cells
ns
0
1
2
3
4
T
T+Zn

c

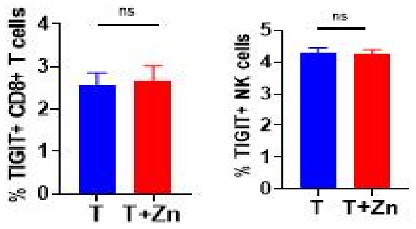
% TIGIT+ CD8+ T cells
ns
0
1
2
3
4
T
T+Zn
% TIGIT+ NK cells
ns
0
1
2
3
4
5
T
T+Zn

d

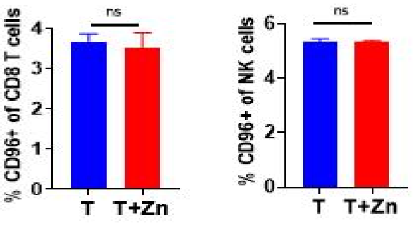
% CD96+ of CD8 T cells
ns
0
1
2
3
4
T
T+Zn
% CD96+ of NK cells
ns
0
2
4
6
T
T+Zn

Fig. S8

a

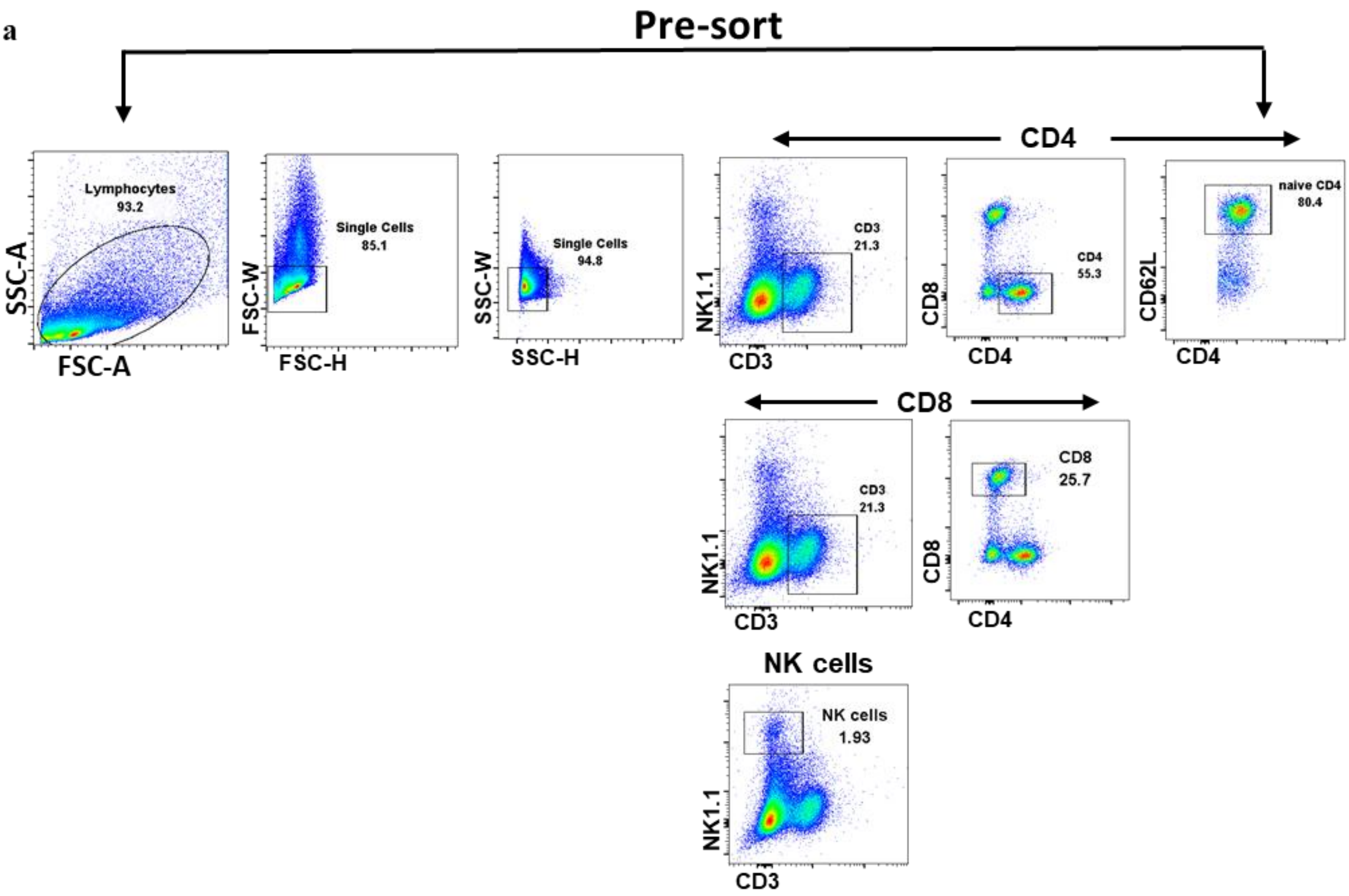

Pre-sort
Lymphocytes
93.2
SSC-A
FSC-A
Single Cells
85.1
FSC-W
FSC-H
Single Cells
94.8
SSC-W
SSC-H
CD4
CD3
21.3
NK1.1
CD3
CD4
55.3
CD8
CD4
naive CD4
80.4
CD62L
CD4
CD8
CD3
21.3
NK1.1
CD3
CD8
25.7
CD8
CD4
NK cells
NK cells
1.93
NK1.1
CD3

b

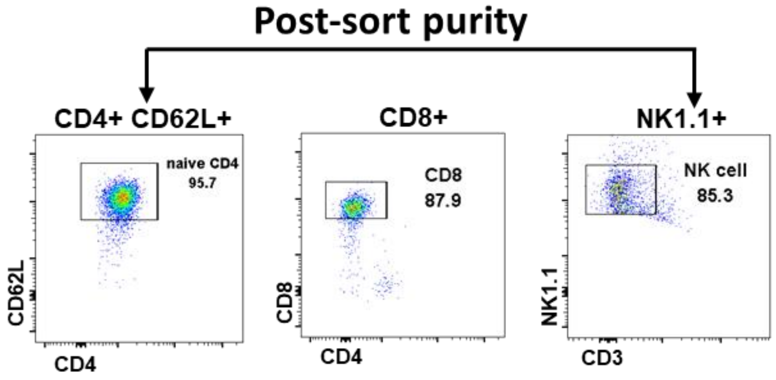

Post-sort purity
CD4+ CD62L+
naive CD4
95.7
CD62L
CD4
CD8+
CD8
87.9
CD8
CD4
NK1.1+
NK cell
85.3
NK1.1
CD3

Fig. S9.

A

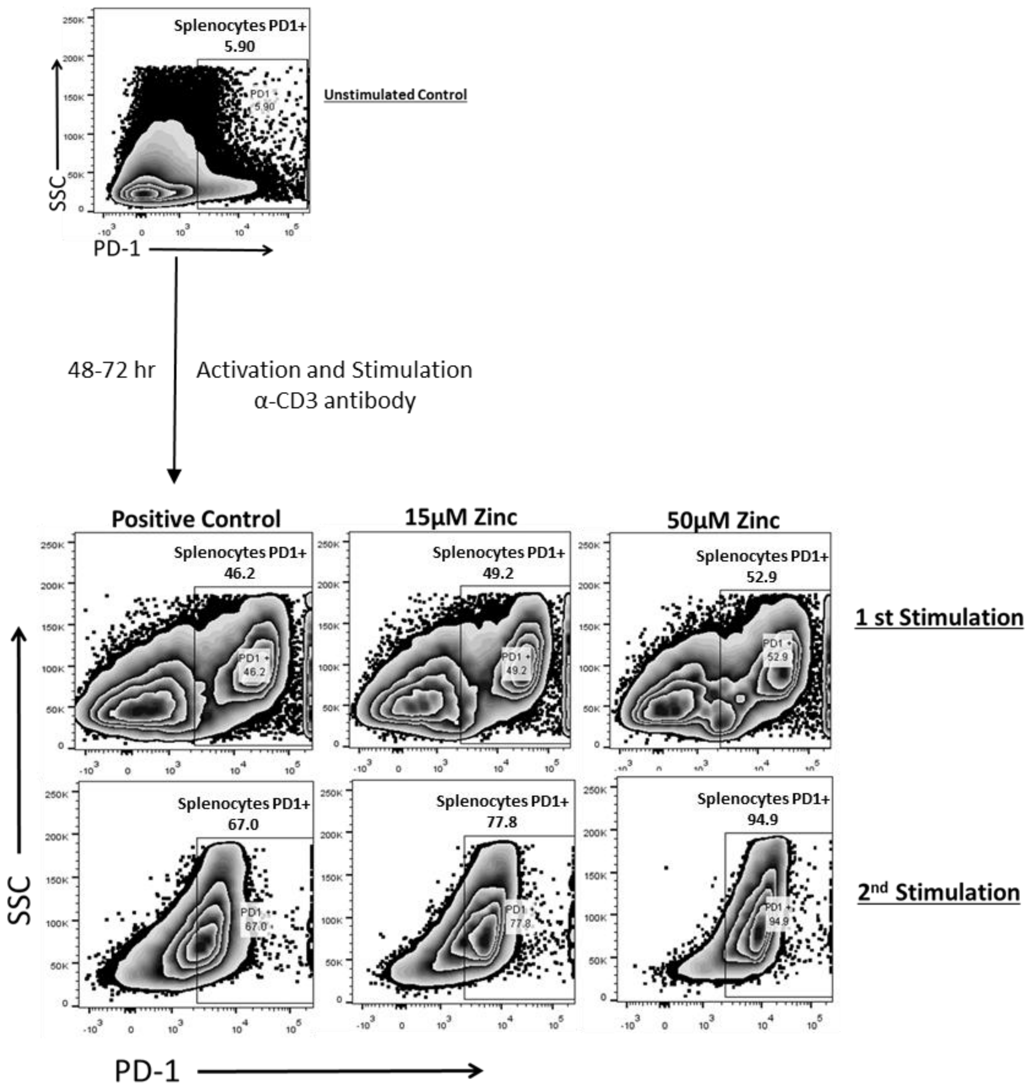
Splenocytes PD1+
5.90
Unstimulated Control
SSC
PD-1
48-72 hr
Activation and Stimulation
α-CD3 antibody
Positive Control
15µM Zinc
50µM Zinc
Splenocytes PD1+
46.2
Splenocytes PD1+
49.2
Splenocytes PD1+
52.9
1 st Stimulation
Splenocytes PD1+
67.0
Splenocytes PD1+
77.8
Splenocytes PD1+
94.9
2nd Stimulation
SSC
PD-1

B

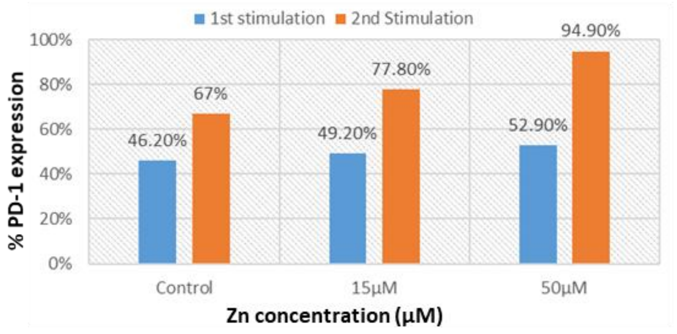
1st stimulation
2nd Stimulation
100%
80%
60%
40%
20%
0%
% PD-1 expression
46.20%
67%
49.20%
77.80%
52.90%
94.90%
Control
15µM
50µM
Zn concentration (µM)

Fig. S10

a

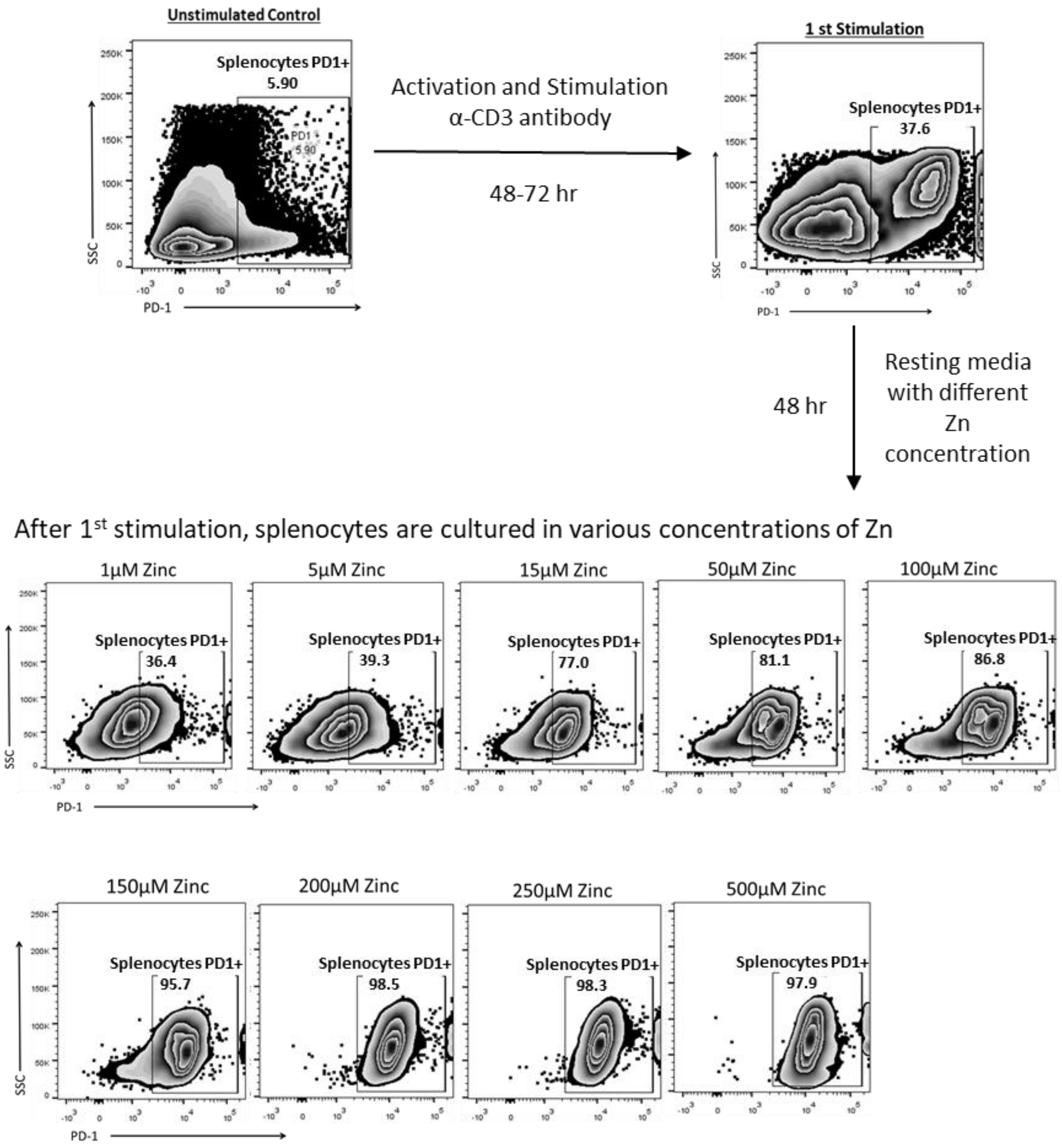

Unstimulated Control
Splenocytes PD1+
5.90
Activation and Stimulation
α-CD3 antibody
48-72 hr
1 st Stimulation
Splenocytes PD1+
37.6
48 hr
Resting media with different Zn concentration
After 1st stimulation, splenocytes are cultured in various concentrations of Zn
1μM Zinc
Splenocytes PD1+ 36.4
5μM Zinc
Splenocytes PD1+ 39.3
15μM Zinc
Splenocytes PD1+ 77.0
50μM Zinc
Splenocytes PD1+ 81.1
100μM Zinc
Splenocytes PD1+ 86.8
150μM Zinc
Splenocytes PD1+ 95.7
200μM Zinc
Splenocytes PD1+ 98.5
250μM Zinc
Splenocytes PD1+ 98.3
500μM Zinc
Splenocytes PD1+ 97.9
SSC
PD-1

b

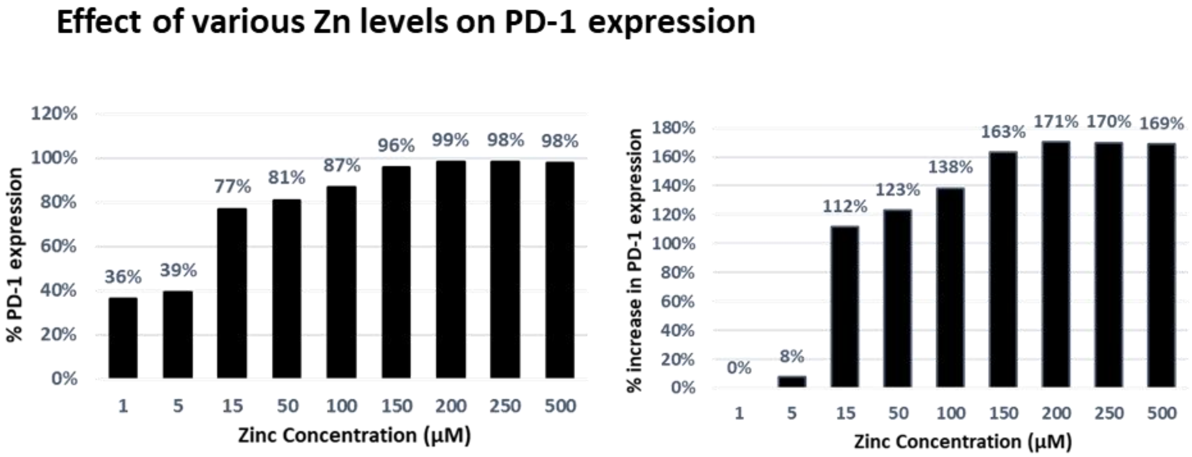

Effect of various Zn levels on PD-1 expression
% PD-1 expression
36% 39% 77% 81% 87% 96% 99% 98% 98%
1 5 15 50 100 150 200 250 500
Zinc Concentration (μM)
% increase in PD-1 expression
0% 8% 112% 123% 138% 163% 171% 170% 169%
1 5 15 50 100 150 200 250 500
Zinc Concentration (μM)

Fig. S11

a

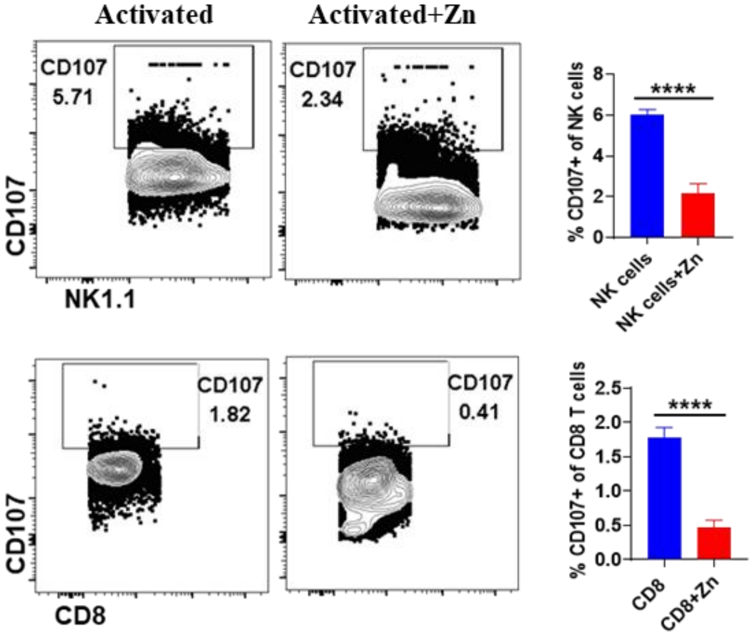

Fig. S12

a

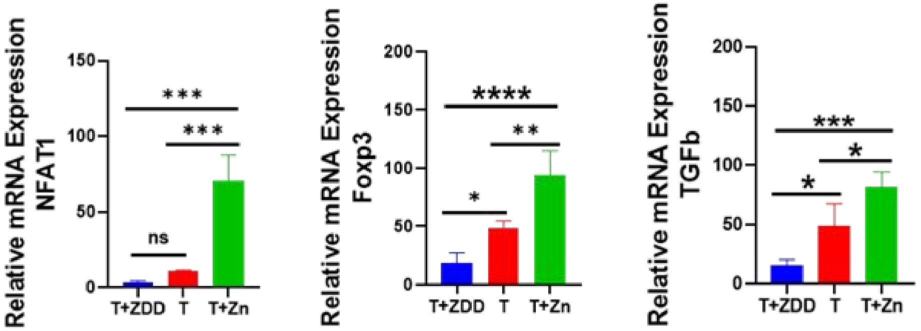

Fig. S13

a

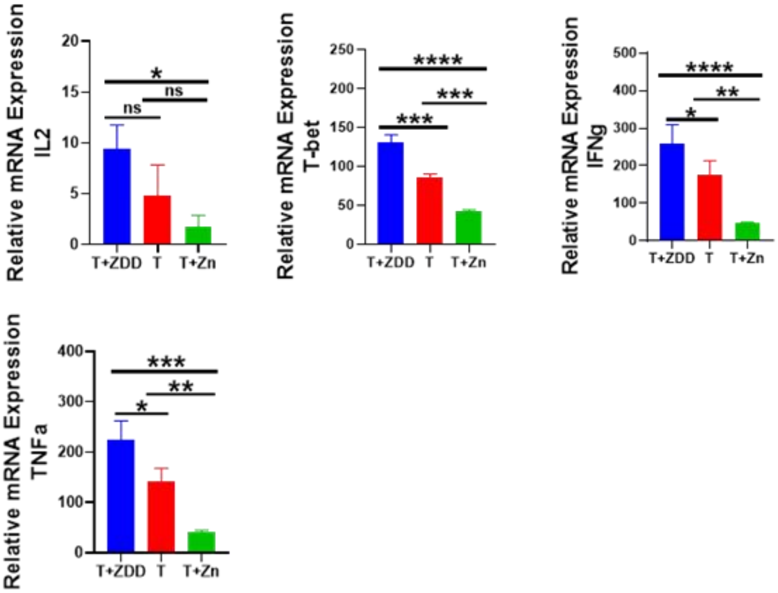

Fig. S14

a

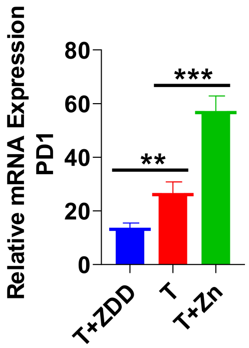

Fig. S15

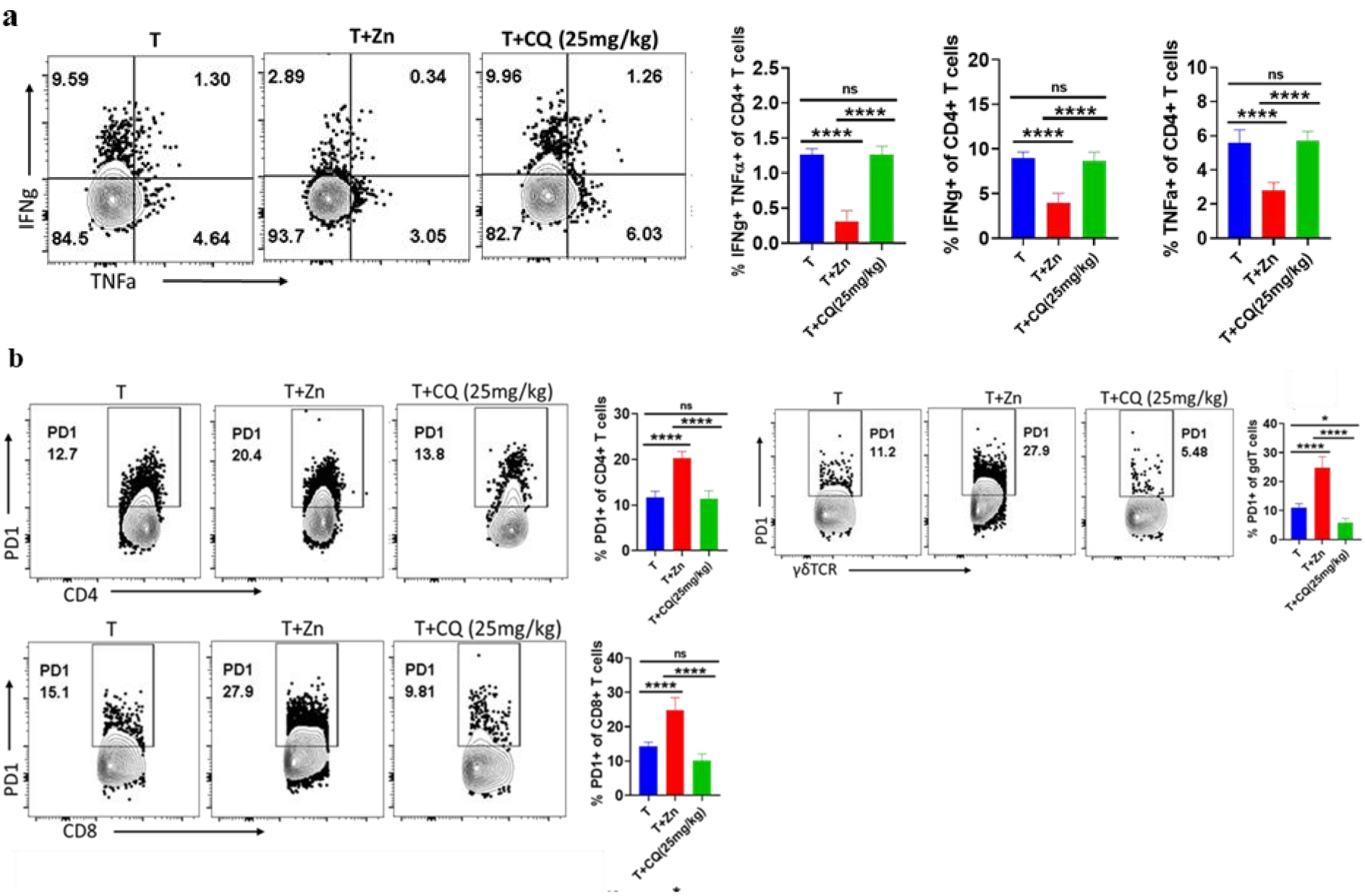

Fig. S16

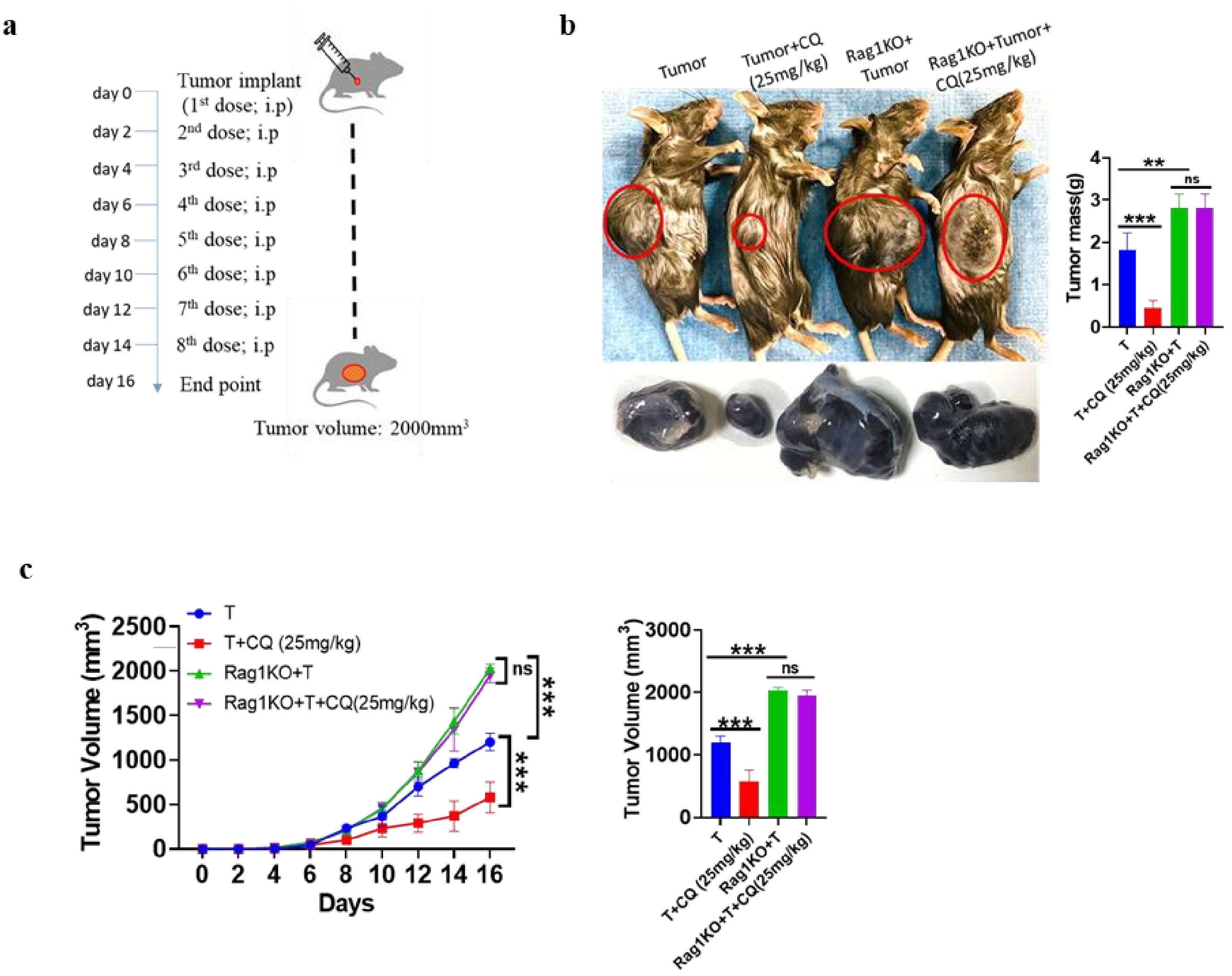
a
day 0
day 2
day 4
day 6
day 8
day 10
day 12
day 14
day 16
Tumor implant
(1st dose; i.p)
2nd dose; i.p
3rd dose; i.p
4th dose; i.p
5th dose; i.p
6th dose; i.p
7th dose; i.p
8th dose; i.p
End point
Tumor volume: 2000mm3
b
Tumor
Tumor+CQ (25mg/kg)
Rag1KO+ Tumor
Rag1KO+Tumor+ CQ(25mg/kg)
Tumor mass(g)
**
***
ns
T
T+CQ (25mg/kg)
Rag1KO+T
Rag1KO+T+CQ(25mg/kg)
c
T
T+CQ (25mg/kg)
Rag1KO+T
Rag1KO+T+CQ(25mg/kg)
Tumor Volume (mm3)
Days
ns
***
***
Tumor Volume (mm3)
***
***
ns
T
T+CQ (25mg/kg)
Rag1KO+T
Rag1KO+T+CQ(25mg/kg)

Fig. S17

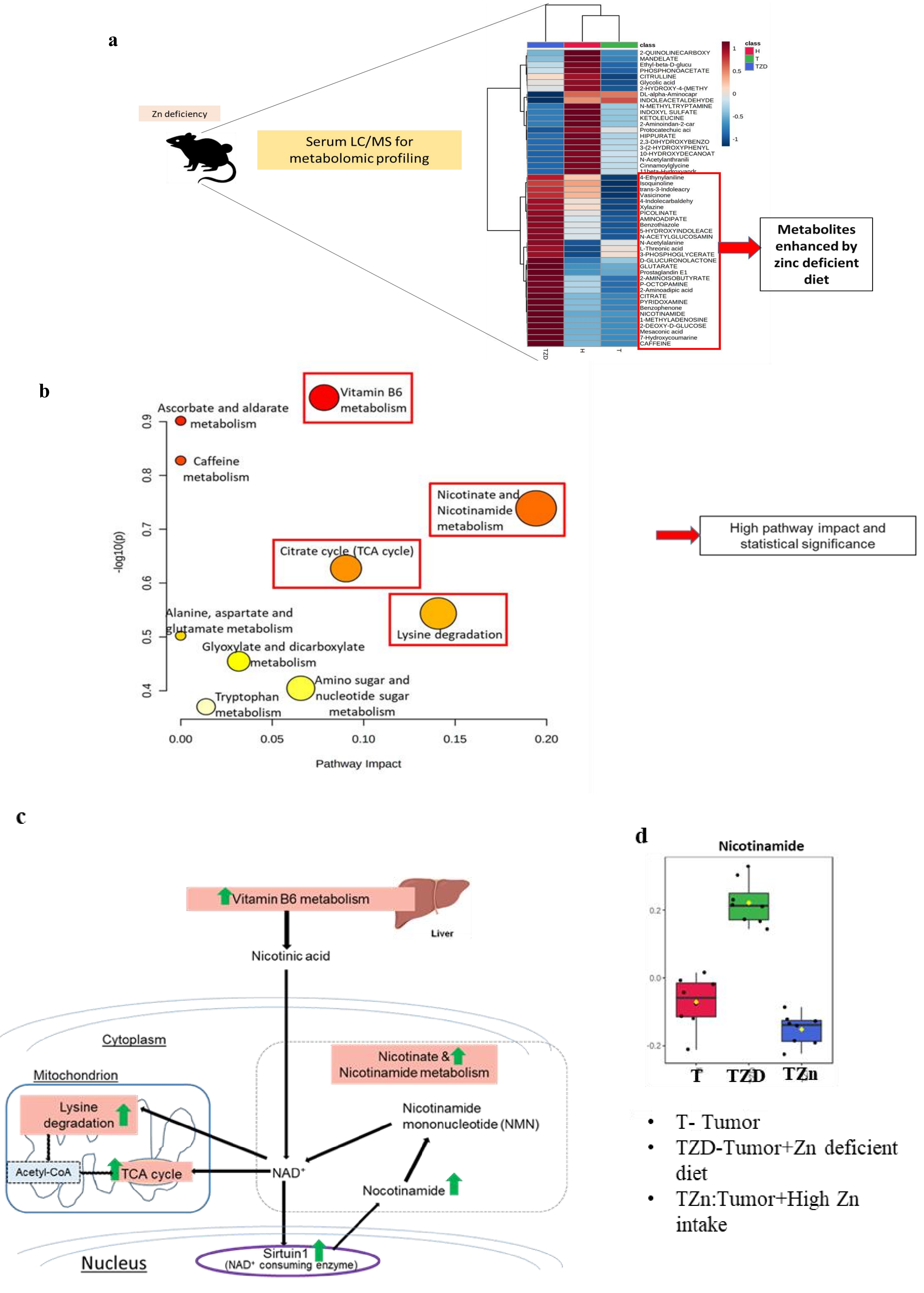

a
Zn deficiency
Serum LC/MS for metabolomic profiling
class
2-QUINOLINECARBOXY
MANDELATE
Ethyl-beta-D-glucu
PHOSPHONOACETATE
CITRULLINE
Glycolic acid
2-HYDROXY-4-(METHY
DL-alpha-Aminocapr
INDOLEACETALDEHYDE
N-METHYLTRYPTAMINE
INDOXYL SULFATE
KETOLEUCINE
2-Aminoindan-2-car
Protocatechuic aci
HIPPURATE
2,3-DIHYDROXYBENZO
3-(2-HYDROXYPHENYL
10-HYDROXYDECANOAT
N-Acetylanthranili
Cinnamoylglycine
4-Ethynylaniline
Isoquinoline
trans-3-Indoleacry
Vasicinone
4-Indolecarbaldehy
Xylazine
PICOLINATE
AMINOADIPATE
Benzothiazole
5-HYDROXYINDOLEACE
N-ACETYLGLUCOSAMIN
N-Acetylalanine
L-Threonic acid
3-PHOSPHOGLYCERATE
D-GLUCURONOLACTONE
GLUTARATE
Prostaglandin E1
2-AMINOISOBUTYRATE
P-OCTOPAMINE
2-Aminoadipic acid
CITRATE
PYRIDOXAMINE
Benzophenone
NICOTINAMIDE
1-METHYLADENOSINE
2-DEOXY-D-GLUCOSE
Mesaconic acid
7-Hydroxycoumarine
CAFFEINE
TZD
H
T
1
0.5
0
-0.5
-1
class
H
T
TZD
Metabolites enhanced by zinc deficient diet
b
-log10(p)
0.4
0.5
0.6
0.7
0.8
0.9
Ascorbate and aldarate metabolism
Vitamin B6 metabolism
Caffeine metabolism
Nicotinate and Nicotinamide metabolism
Citrate cycle (TCA cycle)
Alanine, aspartate and glutamate metabolism
Lysine degradation
Glyoxylate and dicarboxylate metabolism
Amino sugar and nucleotide sugar metabolism
Tryptophan metabolism
0.00
0.05
0.10
0.15
0.20
Pathway Impact
High pathway impact and statistical significance
c
Vitamin B6 metabolism
Liver
Nicotinic acid
Cytoplasm
Mitochondrion
Nicotinate & Nicotinamide metabolism
Lysine degradation
Nicotinamide mononucleotide (NMN)
Acetyl-CoA
TCA cycle
NAD+
Nocotinamide
Nucleus
Sirtuin1
(NAD+ consuming enzyme)
d
Nicotinamide
0.2
0.0
-0.2
T
TZD
TZn
• T- Tumor
• TZD-Tumor+Zn deficient diet
• TZn:Tumor+High Zn intake

Fig. S18

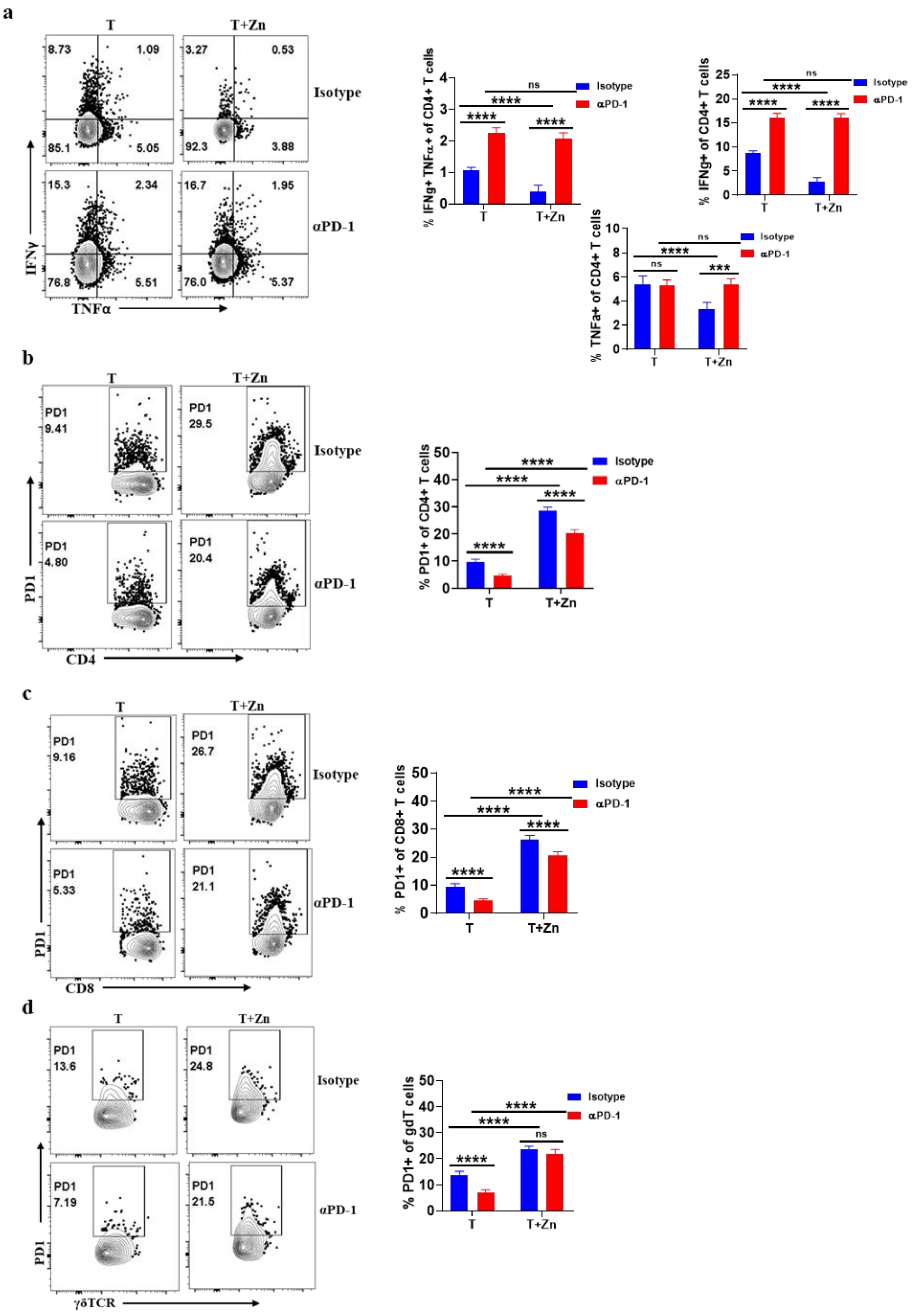

a
T
T+Zn
8.73
1.09
85.1
5.05
3.27
0.53
92.3
3.88
Isotype
15.3
2.34
76.8
5.51
16.7
1.95
76.0
5.37
αPD-1
IFNγ
TNFα
% IFNg+ TNFα+ of CD4+ T cells
% IFNg+ of CD4+ T cells
% TNFa+ of CD4+ T cells
Isotype
αPD-1
ns
****
***
b
PD1
9.41
29.5
4.80
20.4
CD4
% PD1+ of CD4+ T cells
c
PD1
9.16
26.7
5.33
21.1
CD8
% PD1+ of CD8+ T cells
d
PD1
13.6
24.8
7.19
21.5
γδTCR
% PD1+ of gdT cells
